# Skin-Friendly Large Matrix Iontronic Sensing Meta-Fabric for Spasticity Visualization and Rehabilitation Training via Piezo-Ionic Dynamics

**DOI:** 10.1007/s40820-024-01566-3

**Published:** 2024-12-19

**Authors:** Ruidong Xu, Tong Xu, Minghua She, Xinran Ji, Ganghua Li, Shijin Zhang, Xinwei Zhang, Hong Liu, Bin Sun, Guozhen Shen, Mingwei Tian

**Affiliations:** 1 Research Center for Intelligent and Wearable Technology, College of Textiles and Clothing, State Key Laboratory of Bio-Fibers and Eco-Textiles, Health&Protective Smart Textile Research Center of Qingdao, Qingdao University, Qingdao, 266071 People’s Republic of China; 2 Academy of Arts & Design of Qingdao University, Qingdao, 266071 People’s Republic of China; 3 College of Electronics and Information, Qingdao University, Qingdao, 266071 People’s Republic of China; 4 School of Integrated Circuits and Electronics, Beijing Institute of Technology, Beijing, 100081 People’s Republic of China

**Keywords:** Skin-friendly, Large matrix, Iontronic meta-fabric, Spasticity visualization rehabilitation training

## Abstract

Rehabilitation training is believed to be an effectual strategy that can reduce the risk of dysfunction caused by spasticity. However, achieving visualization rehabilitation training for patients remains clinically challenging. Herein, we propose visual rehabilitation training system including iontronic meta-fabrics with skin-friendly and large matrix features, as well as high-resolution image modules for distribution of human muscle tension. Attributed to the dynamic connection and dissociation of the meta-fabric, the fabric exhibits outstanding tactile sensing properties, such as wide tactile sensing range (0 ~ 300 kPa) and high-resolution tactile perception (50 Pa or 0.058%). Meanwhile, thanks to the differential capillary effect, the meta-fabric exhibits a “hitting three birds with one stone” property (dryness wearing experience, long working time and cooling sensing). Based on this, the fabrics can be integrated with garments and advanced data analysis systems to manufacture a series of large matrix structure (40 × 40, 1600 sensing units) training devices. Significantly, the tunability of piezo-ionic dynamics of the meta-fabric and the programmability of high-resolution imaging modules allow this visualization training strategy extendable to various common disease monitoring. Therefore, we believe that our study overcomes the constraint of standard spasticity rehabilitation training devices in terms of visual display and paves the way for future smart healthcare.

**Supplementary Information:**

The online version contains supplementary material available at 10.1007/s40820-024-01566-3.

## Highlights

- The iontronic meta-fabric exhibits a “hitting three birds with one stone” property, breaking through the bottleneck that traditional film materials (PDMS) cannot balance comfort and durability.
- The meta-fabrics can be integrated with garments and advanced data analysis systems to manufacture a series of large matrix structure (> 40 × 40, 1600 sensing units) rehabilitation training devices, overcoming the bottleneck of low matrix integration of traditional iontronic devices (< 10 × 10, 100 sensing units).

**Supplementary Information:**

The online version contains supplementary material available at 10.1007/s40820-024-01566-3.

## Graphical abstract

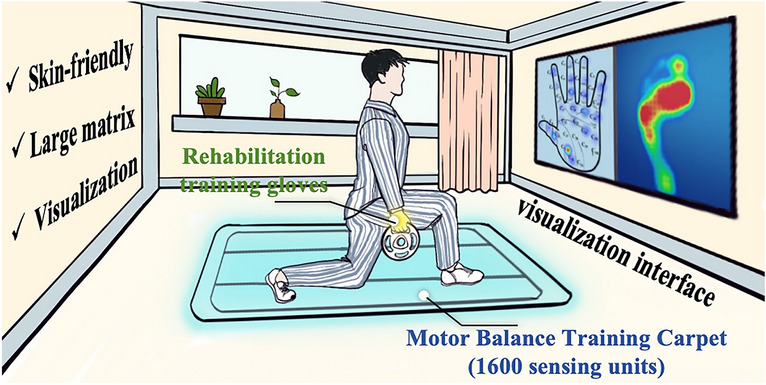

## Introduction

As a common sequel of central nervous system injuries such as such as stroke, traumatic brain injury (TBI) and cerebral palsy (CP), spasticity has now been a globally prevalent and disabling disease [1, 2]. It is estimated that over millions people worldwide suffer from spasticity to some degree [3]. Take the spasticity caused by stroke as an example, it occurs in 25% of patients within 2 weeks after a stroke, and increases up to 43%–50% and 38%–44% after 6 and 12 months, respectively [4–6]. Among them, severe or disabling spasticity occurs in approximately 15% of stroke patients, which is caused by increasing in muscle tone [7, 8]. Fortunately, rehabilitation training, which can improve muscle contraction, coordination, and control abilities, is seen as an effective strategy to avoid the risk of disability. To fulfill this effect, the assessment of muscle tone is of a significant importance.

Currently, mainstream strategies for muscle tension assessment, such as Modified Ashworth scale (MAS) and Modified Tardieu scales (MTS), are testing joint resistance of the patients during passive motion [2, 9–11]. Nevertheless, the above strategies rely heavily on the subjective perception of the physician, which may lead to potential misdiagnosis [12]. In addition, various quantitative assessment techniques, such as electrophysiological measures, gait analysis and neuroimaging, have emerged in clinical in recent years [13–15]. These techniques are dependent on various bulky and costly medical apparatus, which not only require professional operators but cause uncomfortable experience for patients [16, 17]. Thus, it is difficult for physician to obtain timely and continuous rehabilitation training data for patients, affecting the recovery of patients. To this end, portable muscle tension monitoring technologies with continuous monitoring and wearing comfort are highly demanded.

Thanks to the advent and development of flexible electronics, wearable tactile sensing devices (such as resistance, capacitance, triboelectric, and iontronic type) have provided a technological capability to dynamically monitor muscle tone (continuous monitoring time > 24 h) through pressure-induced force-electricity mapping conversion, demonstrating great potential in the field of rehabilitation training [18–26]. Among these devices, iontronic-type tactile sensing devices have drawn much interests in recent year. Under the stimulation of an AC electric field, the iontronic tactile sensing device can trigger ultra-high capacitance per unit area (> 10 µF cm^−2^) due to the nanoscale electronic double structure (EDL) formed at the iontronic interface. Compared with traditional capacitive devices (< 1 nF cm^−2^), the capacitance signal of the iontronic tactile sensor is improved by more than 1000-folds, essentially eliminating the interference of parasitic noise and environmental electromagnetic field on the sensing signal of devices [27–30]. To improve the tactile sensing performance, structured ionic conductor (such as pyramid, pillars and hemisphere) has often been fabricated to increase the contact area of the iontronic interface under pressure loading [31–33]. However, the above structures usually require sophisticated fabrication techniques such as 3D printing or two-photon laser, resulting in poor matrix integration (< 10 × 10, 100 sensing units) [34–36]. This drawback restricts the application of these devices in refined rehabilitation training. In addition, the visualization system has not been integrated into such devices, posing challenges to the realization of digital rehabilitation training. Therefore, it is imperative to develop wearable iontronic devices with large matrix and visualization functions.

Herein, we construct a closed-loop spasticity visualization rehabilitation training system composed of iontronic meta-fabric with skin-friendly and large matrix properties and multi-channel programmable data analysis modules. Specifically, we employed dome-shaped polyacrylamide-lithium chloride (PAAM-LiCl) ionic hydrogel and conductive knitted fabric to construct iontronic meta-fabric. After pressure-induced deformation of the dome-shaped ionic hydrogel, the process of dynamic connection and dissociation at the iontronic interface are exacerbated, resulting in wide range tactile sensing range (0 ~ 300 kPa) and high-resolution tactile perception (50 Pa or 0.058%). Meanwhile, the meta-fabric exhibits a “hitting three birds with one stone” property (dryness wearing experience, long working time and cooling sensing, respectively) due to the differential capillary effect. Notably, the meta-fabrics can be seamlessly integrated with garments to fabricate series of large matrix structures (> 40 × 40, 1600 sensing units) rehabilitation devices such as grip strength training gloves and motor balance training carpet. Meanwhile, well-trained data analysis modules are integrated to achieve real-time visual color parsing of muscle tone for patients. Notably, this approach can be expanded to various common disease monitoring, demonstrating excellent generality and versatility. This study provides insight into visualization tactile sensing devices and paves the way for future smart healthcare.

## Experimental Section

### Materials

Graphene solid slurry (10 wt%, lateral size 20 μm) was purchased from Ningbo Moxi Co. Ltd (China). Acrylamide (AAM) and lithium chloride (LiCl) were purchased from ALADDIN Co. Ltd. Ammonium persulfate (APS) and N, N-methylene bisacrylamide (MBAA) were purchased from Sigma-Aldrich Co. Ltd (American). N, N, N’, N-tetramethylethylenediamine (TEMED) was purchased from ALFA Co. Ltd. PE/PP core-spun yarn (5.64 tex) and PBT yarn (5.64 tex) were purchased from Lutai Textile Co.Ltd (China). Commercial fabrics (cotton knitted fabric, cotton woven fabric, PET woven fabric and PET non-woven fabric) were purchased from Furi Textile Co. Ltd (China).

### Preparations

#### Preparation of the Programmable PAAM-LiCl Ionic Hydrogel

The PAAM-LiCl ionic hydrogel was synthesized following a previously reported method with slight modifications [61]. Briefly, AAM power (2 M, monomer), LiCl (3 M, conductive material), APS (0.16 wt%, initiator) and MBAA (0.006 wt%, crosslinker) were dispersed in deionized water and stirred at room temperature for up to 1 h to obtain a homogeneous solution. Then, TEMED (0.25 wt%, accelerator) is added to the above solution. The precursor solution is poured into acrylic molds with three different structures (plane, corrugation and dome shape) and then thermally polymerized under 80 °C for 20 min to obtain pattern ionic hydrogels.

#### Preparation of the Weft-Knitted Double-Side Jacquard Fabric

Firstly, the PE/PP core-spun yarn and the PBT yarn were unwound on the upper and lower needle beds of the circular knitting machine respectively by the drafting rollers. Subsequently, the fabric structure is changed on the basis of 1 + 1 rib knitting. Specifically, the PE/PP core-spun yarns were wound in alternate loops on the upper and lower needle beds to form the lower and intermediate connecting layers of the double knitted fabric. The PBT yarns were mainly looped in the lower needle bed to form the top layer of the double knitted fabric.

#### Preparation of the Skin-Friendly Iontronic Meta-Fabric

The pristine double-side jacquard fabric was cleaned with deionized water (DI) by ultrasonic cleaner and dried in a vacuum oven (70 °C, 30 min). Then, the graphene slurry (solid content of 10 wt%) was placed into a dispenser for high-pressure dispersion (85 MPa) and repeated three times to obtain a uniformly dispersed graphene aqueous solution. Then, the jacquard fabric was repeatedly immersed into uniformly graphene aqueous solution at room temperature for 10 min and dried in a vacuum oven to obtain conduction (80 °C, 30 min). Finally, the pattern ionic hydrogels were encapsulated by two layers of knitted fabric to fabricate the tactile sensorimotor interface.

### Design of Closed-Loop Spasticity Visualization Training System

The closed-loop spasticity visualization training system consists of a pair of tactile monitoring glove, lower-level circuit, and upper-level software. The system collects signals through the monitoring glove and lower-level circuit and sends them to the upper-level software through a serial port. The upper-level software performs data analysis, display, and other functions.

#### Design of the Lower-Level Circuit

The circuit board uses a main control board manufactured by STMicroelectronics (STM32F407VET6), including 3 × 12-bit analog-to-digital converters (ADC), 2 digital-to-analog converters, and a real-time clock (RTC). Among them, Max4871 is used as the analog switch in the circuit. Depending on the logic level signals of A and B inputs (00, 01, 10, 11), the X_0_ ~ X_4_ and Y_0_ ~ Y_4_ ports are used as inputs for the Max4871. By controlling the A and B inputs, a selected signal can be routed to the X and Y outputs. The output signals are then converted into analog signals through the ADC0. The serial communication standard used for the output is RS-485, and the max485 chip is employed to convert the transistor–transistor logic level signals (TTL) generated by the lower-level circuit into RS-485 levels to comply with the RS-485 communication protocol. Lastly, the transmission of digital signals is achieved through a DB9 data interface connector. This circuit exhibits various outstanding properties such as low power consumption, a large number of interface pins, high accuracy, and fast data acquisition speed, meeting the signal acquisition requirements for this work.

#### Serial Communication and Reset Circuit Design

This work implements the data communication function utilizing serial communications. The QtSerialPort class is used to set serial port settings such as baud rate, stop bits, parity, and data bits, which correspond to the serial port parameters in lower-level circuits for signal transmission. The available serial ports are updated in real time by refreshing the serial port list, which eliminates the problem of having only one serial port. The reset circuit consists a resistor and a capacitor, which are controlled by the upper-level software to reset the circuit after testing is stopped.

#### Design of Visualization Rehabilitation Training Interface

Qt Design software is used to design the interface system, which enables parsing and displaying of the collected data. The interface is designed using various controls such as PushButton, Widget, and Label from the interface design module of Qt. The QPainter class is used to create a canvas as the base for all graphics displays. The data transmitted from the lower-level circuit are color-coded based on its numerical value using the QColor class. The QRadialGradient class is utilized to control the color area of each data point, and a threshold is set to filter out abnormal and noisy data, ensuring a clear visualization of the data graph and reducing signal interference issues during graphic display.

### Characterization

Morphological analysis of samples was accessed based on scanning electron microscopy (SEM, TESCAN VEGA3, Gzech) images. The change of currents was tested using desktop multi-meters (Keysight 34461A, Agilent, USA). The AC power are applied by a function generator (GA 1651A, Atten, China). The uniformly dispersed graphene solution was obtained by a nano-homogenize machine (AH-2010, ATS Engineering Limited, China). The freeze-drying machine obtains dehydrated ionic hydrogel (Alpha 2-4LSC BASIC, Christ, Germany). The quick-drying property was tested by MMT liquid moisture management tester (G290, Qinsun, China). The cooling sensing was tested by a thermal resistance meter (CSI-287BB, Chengsi, China). The interaction system was designed by Python programming language. The weft-knitted double-side jacquard fabric was fabricated by the circular knitting machine (TF-S3F4, Taifan, China).

## Results and Discussion

### Construction of the Iontronic Meta-Fabric

**Fig. 1:**
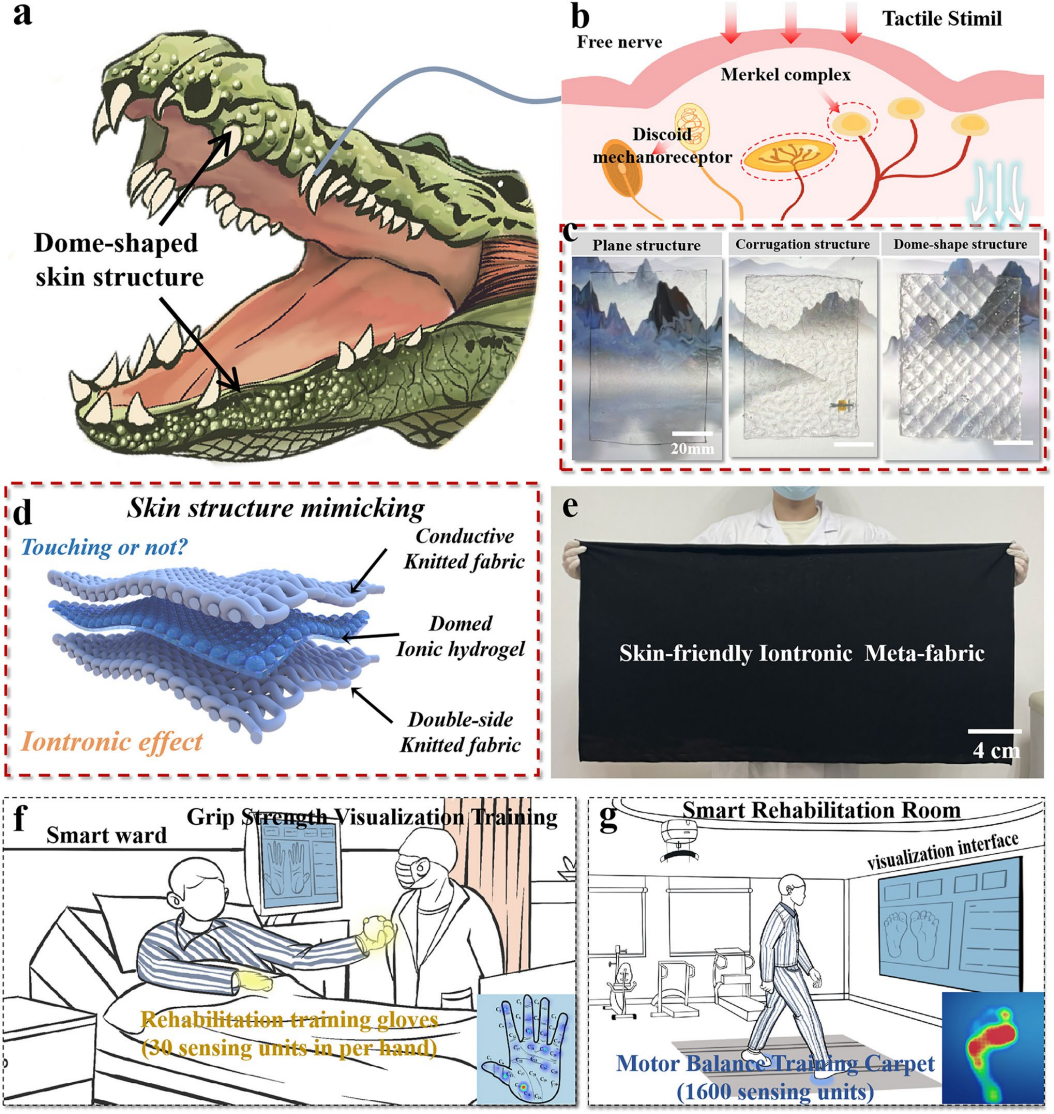
Construction of tactile sensorimotor interface. **a, b** Schematic of the dome-shaped structure on the skin surface of crocodiles. **c** Morphology of structured ionic hydrogel and it microstructure. **d, e** Structural schematic and image of the tactile sensorimotor interface. **f, g** Concept of the spasticity rehabilitation training gloves and carpet

Crocodiles are considered as one of the top predators on the earth, benefitting from the naturally evolved dome-shaped pressure receptors on the skin. The pressure receptors contain complex sensory nervous systems, including free nerve, discoid mechanoreceptor and merkel complex, rendering a precisely transmitting and amplifying of the external stimuli. Therefore, crocodiles are capable of sensing tiny water disturbances caused by prey such as the Tibetan antelope, so that they can carry out accurate predatory behavior (Fig. 1a, b) [37, 38]. Inspired by this unique sensory organ of a crocodile, the general layout of the dome-shaped biological perceptual structure has been introduced in our iontronic meta-fabric. To construct the dome-shaped architecture, PAAM-LiCl solution was firstly injected into a dome-shaped PDMS mold and then placed at 80 °C for 20 min. After completion of the chain polymerization reaction, the dome-shaped transparent PAAM-LiCl ionic hydrogel (bottom diameter: 10 mm, height: 1.5 mm) has been fabricated, exhibiting a 3D porous microstructure (Figs. 1c and S1). This structure results in a superior flexibility and elasticity, which is crucial to the wearable tactile sensing devices. Finally, the resultant ionic hydrogel is sandwiched into two layers of fabrics to construct the prototype of the meta-fabric (Fig. 1d). Interestingly, the method is very simple and straightforward, and the meta-fabric can be fabricated on a large scale (Fig. 1e), demonstrating the potential for real application. As a proof of concept, we propose two concrete scenarios (smart ward and smart rehabilitation room) to demonstrate the practicability of our work, including grip strength training gloves (30 sensing units in per hand) and motor balance training carpet (> 40 × 40, 1600 sensing units), achieving high-resolution analysis of human muscle tension information in real-time (Fig. 1f, g).

### Tactile Sensing Properties of the Iontronic Meta-Fabric

**Fig. 2:**
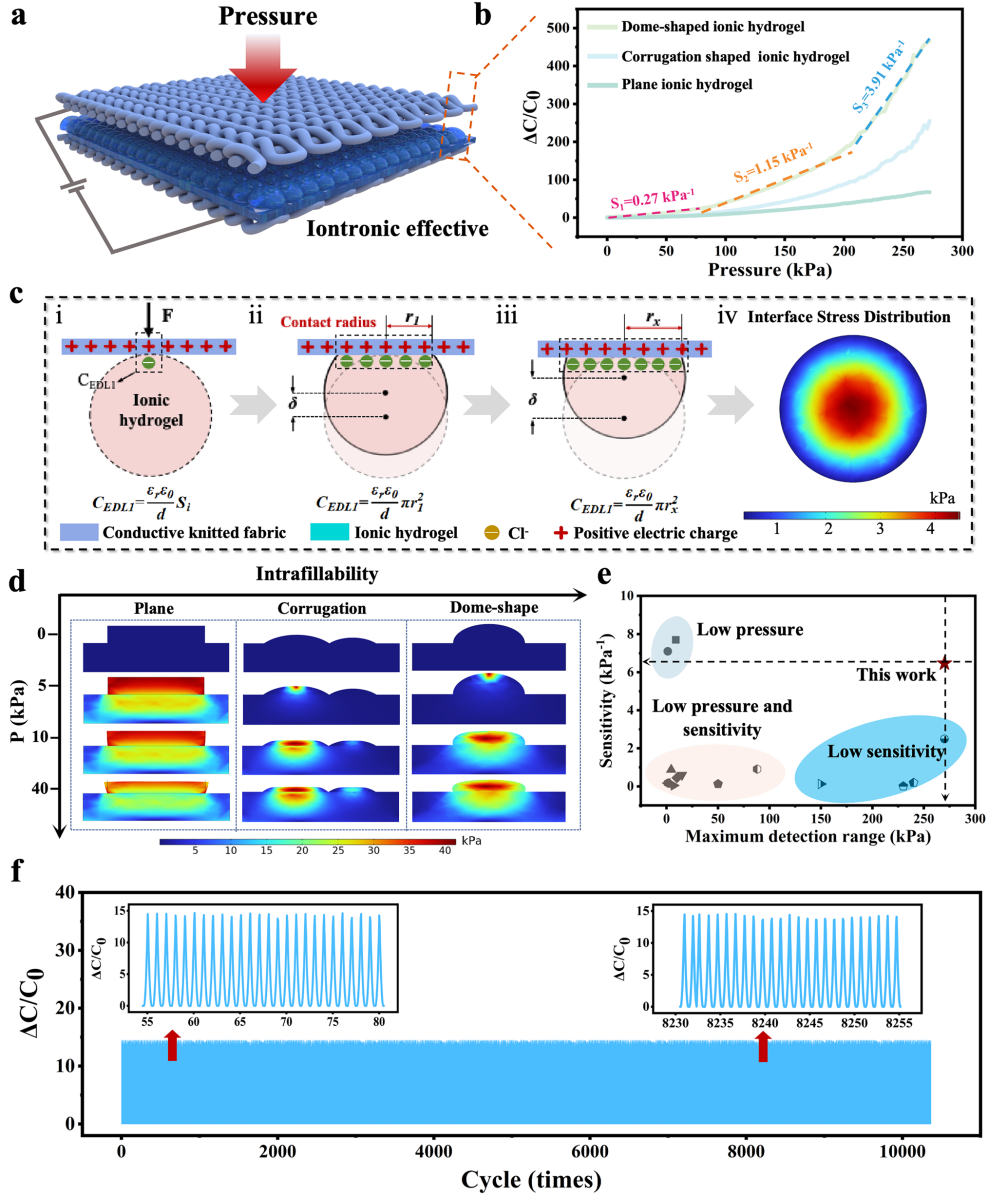
Principles of the programmable meta-fabric design and the sensing mechanism. **a** Change of capacitance over the pressure range up to 270 kPa of the iontronic meta-fabric. **b, c** Sensing mechanism of the meta-fabric before and after applying pressure. **d** Stress distribution of simulation results for different architectures under pressures up to 40 kPa: a plane; a corrugation, and a dome shape. **e** Sensitivity and maximum detection range comparison of previously reported tactile sensors. **f** Mechanical stability of the meta-fabric

As a key parameter to appraise the performance of iontronic-type tactile sensors, the sensitivity is defined as *S* = (Δ*C*/*C*_0_)/Δ*P*, where *C*_0_ is the initial capacitance before pressure loading and Δ*C* is the change in capacitance [21, 39, 40]. The capacitance response of our meta-fabric with dome-shaped ionic hydrogel under pressure is shown in Fig. 2a. One can see that the averaged sensitivity (*S*_1_) is 0.27 kPa^−1^ when the pressure is below 60 kPa, and *S*_2_ is about 1.15 kPa^−1^ within the pressure range of 60–200 kPa. In the high-pressure regime (> 200 kPa), the meta-fabric exhibits a nearly linear response with a sensitivity (*S*_3 _≈ 3.91 kPa^−1^). To further illustrate the excellent tactile sensing property of our meta-fabric, the other morphology of sensing layer (corrugation and plane) have been fabricated. Meanwhile, we have encapsulated the above sensing layers in double-side jacquard fabrics to assemble three meta-fabrics. The sensitivity of the meta-fabric with corrugation active layer shows decreases trend (*S*_1_ = 0.21 kPa^−1^, *S*_2_ = 0.64 kPa^−1^, *S*_3_ = 2.12 kPa^−1^) within the same pressure range. In addition, the meta-fabric with plane active layer displays poor tactile sensing property, the maximum sensitivity is only 0.24 kPa^−1^ (Fig. 2b). The reason can be attributed to the dome-shaped structure fabrication strategy enhancing the iontronic effect between upper layer modification knitted fabrics and the middle active layer in our meta-fabric. In detail, under an AC electric field (< ± 1 V, 20 kHz), the electrons in the knitted fabric attract the counter ions (e.g., Cl^−^) in the active layer, which can construct the electric double-layer structure (EDL) at the nanoscale level (Figs. 2c(i) and S2). Thus, when the dome-shape structure of the middle active layer is compressed under external pressure loading, a high increase in the contact area between the upper fabric and the active layer occurs. It causes the amount of charge induced by the EDL (*C*_EDL1_) to exhibit an increasing trend, resulting in a significant rise in the capacitive signal of our device (Figs. 2c(ii-iii) and S3). It is also verified by the pressure distribution at the cross-section of the ionic hydrogel, which clearly demonstrates the intensity of the deformation of the ionic hydrogel under external force (Fig. 2c(iv)). Besides, because of the outstanding elasticity of the dome-shape ionic hydrogel, the morphology of the active layer can rapidly recover to its original state when the external pressure loading is withdrawn (Fig. S4).

In order to deeply perform the underlying mechanism of the remarkably sensitivity over a broad pressure regime, the meta-fabric with the three active layer structures were analyzed by finite element analysis (FEA), respectively (Fig. 2d). In this course, the device with plane active layer represents obvious pressure-resistance once intimate contact is formed, similar with rigid structures. Therefore, the meta-fabric with a plane active layer remains almost constant over a wide pressure range. The corrugation structure can improve the structural compressibility of the device compared to the plane one, due to the positive Poisson’s ratio of the active layer (PAAM hydrogel) [41]. As external pressure is loaded, the corrugation structure deforms laterally, providing more room for compression than plane structure. However, restricted to the height and irregular morphology of the corrugation structure, it cannot provide more compression space under high pressure, validating the fact that this device has a lower sensitivity at high pressure. While the dome-shaped active layer has a homogeneous morphology and higher geometric height, which contributes to more room for the large and uniform lateral deformation under external pressure, than that of the former two structures, before it fully contact the bottom (Fig. 2d, from 5 to 40 kPa). In addition, our meta-fabric exhibits advantages in terms of tactile sensing sensitivity and working range compared to other relative works [42–57] (Fig. 2e). Besides, the fast response and release speed (47 and 53 ms, Fig. S5) has been achieved due to the precise designed structure, which is beneficial to real-time signal transmitting in applications. Moreover, high mechanical durability over a long time is another core parameter to assess the properties of tactile sensors. Under repeated compression/release test of more than 10,000 cycles with a pressure of 25 kPa, our sensor exhibits a superior stability, and no signal drift or fluctuation appears, as shown in Fig. 2f, which is attributed to the excellent mechanical bonding of ionic hydrogel interface (Figs. S6–S9).

### High Tactile Sensing Resolution of the Iontronic Meta-Fabric

**Fig. 3:**
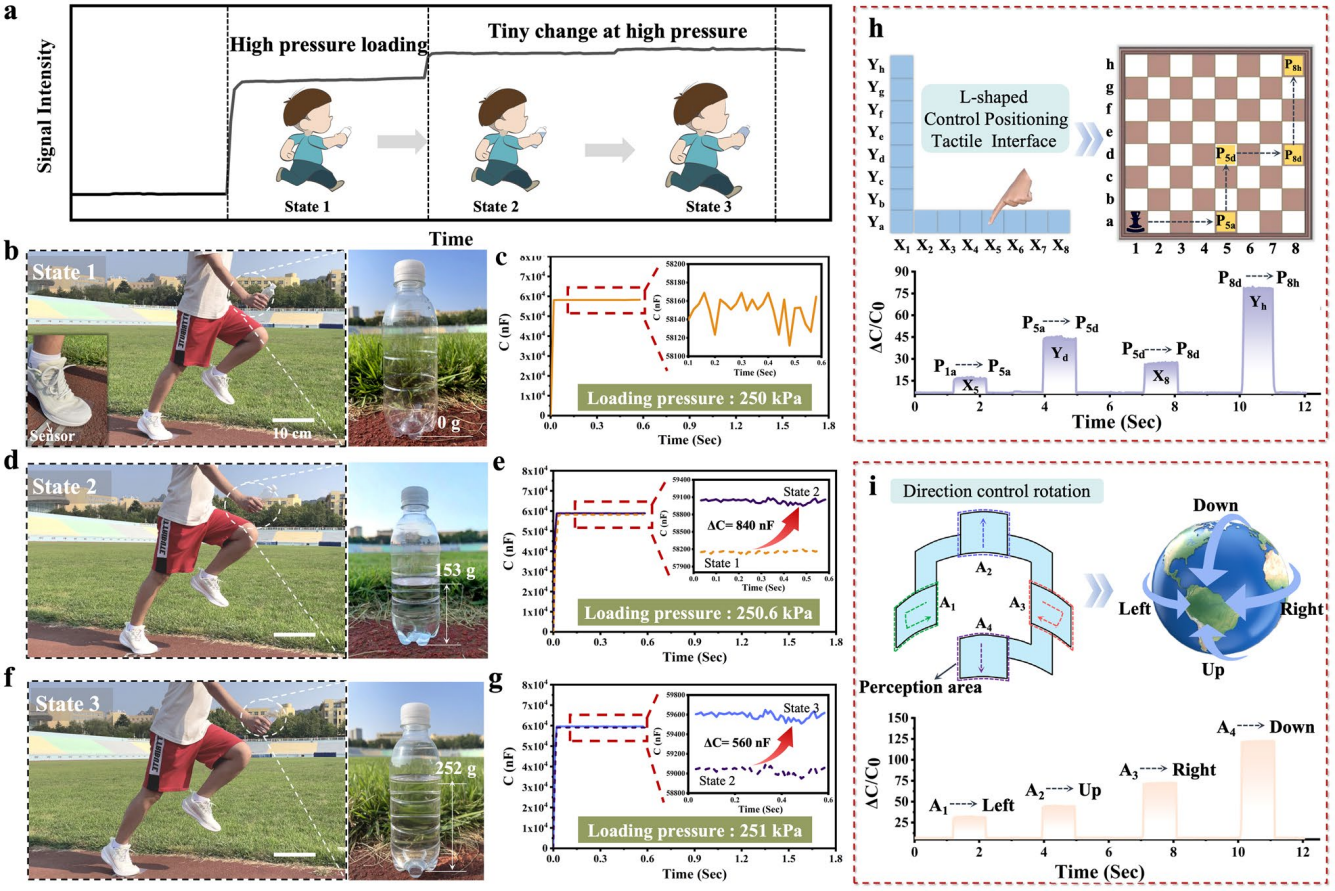
High tactile sensing resolution of the meta-fabric. **a** Schematic illustration of the response of the iontronic tactile sensing to low and high pressure, and detection of micro pressure under high pressure. **b-g** Detection of different micro pressure loading (600 and 400 Pa) applied to the tactile sensing network. **h**, **i** The interactive application of the meta-fabric array

Due to the dome-shape architecture, our meta-fabric possesses two essential features of ideal tactile sensors: remarkable stability and high-sensing resolution (Figs. 3a and S10, S11). To further investigate the potential for real application of our meta-fabric, a square-shape tactile sensing device (50 × 50 mm^2^) was prepared to detect the external pressure loading, and a tester (~ 63 kg) holding an empty plastic bottle stepped over the device, i.e., a reference pressure (~ 250 kPa) was applied to the sensing unit (State 1, Fig. 3b). From Fig. 3c, it can be seen that the capacitance of the sensing unit sharply increase from 181.25 to 58,100 nF once a pressure loading of 250 kPa is applied to the device, indicating that our device is sensitive to high pressure loading. When the tester holding the bottle (containing 153 g water) again stepped over the device (ΔP ~ 600 Pa) (State 2, Fig. 3d), the corresponding capacitance is changed into about 58,900 nF. Namely, the capacitance of the device is improved by about 840 nF compare to state 1 (Fig. 3e and inset graph). As the bottle weight increased to 253 g (Δ*m* = 102 g) (State 3 of Fig. 3f), the pressure applied to the device increases by 400 Pa compared to state 2, resulting in a capacitance of approximately 59,620 nF, 560 nF higher than state 2 (Fig. 3g and inset graph). In addition, under a reference pressure followed by consecutively applying relatively small pressures, the capacitance demonstrates a stepped stable escalation within a very short response time (Fig. S12a–c). These findings show that our device can precisely detect even small change in pressure changes. As a proof-of-concept, an L-shaped positioning controller made of 15 meta-fabrics was designed by touching the corresponding area (Fig. S13). For example, when a finger presses the X_5_, the capacitive signal generated by the associated meta-fabric is sent to the programming software, which controls the chess movement from P_1a_ to P_5a_ (Fig. 3h). Similarly, when the finger presses additional buttons (Y_d_, X_8_ and Y_h_, respectively), the chess moves from P_5a_ to P_8h_ via P_5d_ and P_8d_. A device capable of achieving direction interaction has been built, consisting of four square-shape meta-fabrics (10 × 10 mm^2^) called A_1_, A_2_, A_3_, and A_4_, respectively (Fig. S14). Pressing the button in different locations causes a difference in the rate of change of capacitance change, and the rotation of the sphere up, down, left and right can be controlled.

### Skin-Friendly Property of the Iontronic Meta-Fabric

Favorable moisture absorbing and quick-drying properties of wearable electronics can prevent human skin from discomfort and irritation during the wearing, playing a vital role in maintaining and regulating the human skin micro-environment [58]. To obtain the meta-fabric with desired wearing comfort, a weft-knitted double-side jacquard fabric has been fabricated as a substrate material to evaluate its moisture absorbing and quick-drying properties. For comparison, four types of commercial fabric (double-side knitted fabric, cotton knitted fabric, cotton woven fabric, PET woven fabric, and PET non-woven fabric) are also introduced (Fig. S15).

**Fig. 4:**
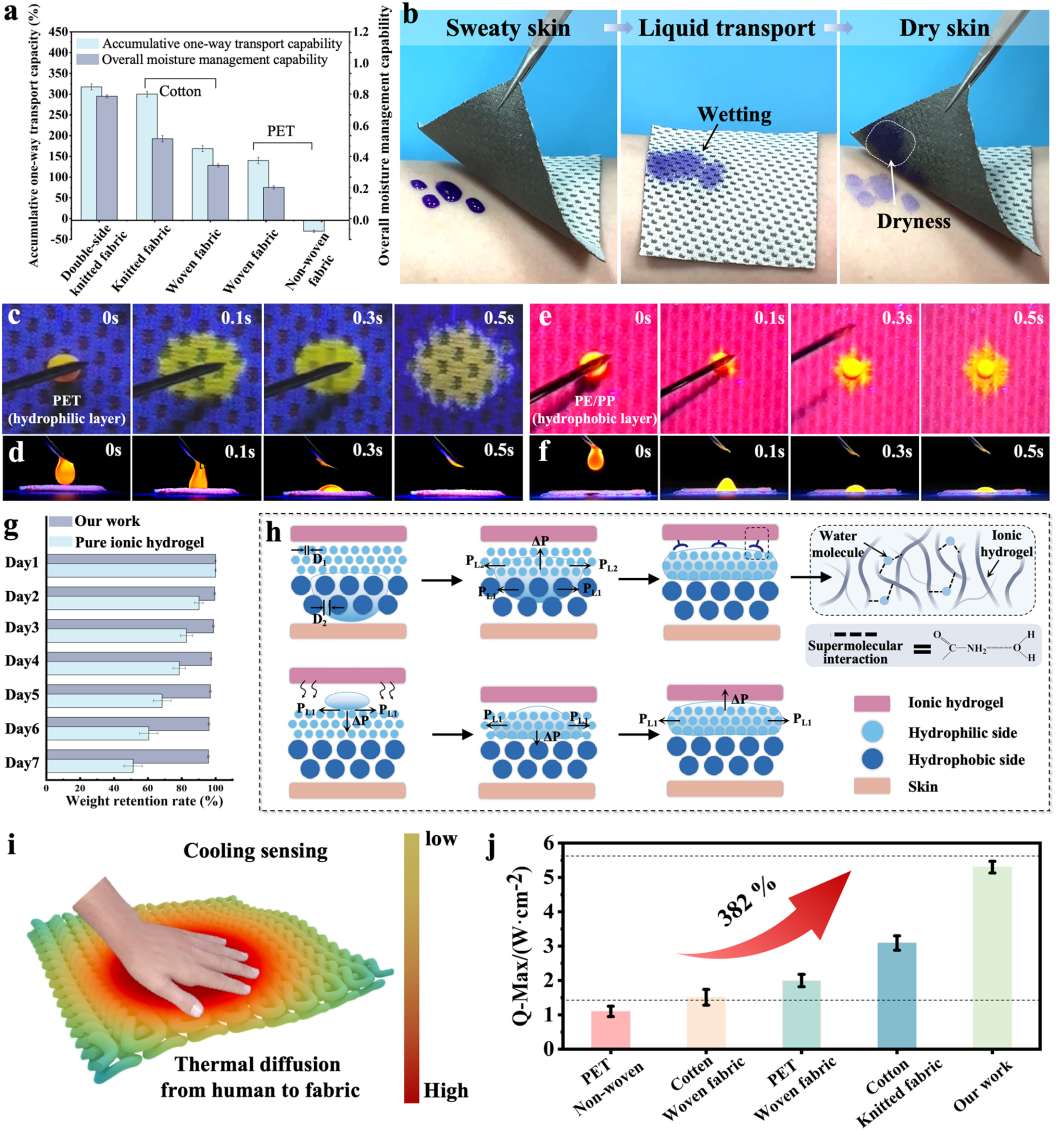
Wearing comfort and differential capillary effect of the meta-fabric.** a** Moisture absorbing and quick-drying properties between our double-side jacquard fabric and other commercial fabrics. **b** Practical application of the double-side jacquard fabric on fast sweat-wicking. Wetting (top view) and unidirectional liquid transport (side view) tests on the **c, d** hydrophilic PBT side (contact with ionic hydrogel) and **e, f** hydrophobic PP/PE side (directly contact with skin). **g** Weight retention rate between our meta-fabric and pure ionic hydrogel. **h** Mechanism of differential capillary effect and supermolecular interaction of our meta-fabric. **i** Schematic illustration of cooling sensing of the meta-fabric. **j** Q-Max value of our meta-fabric

Notably, the accumulative one-way transport capacity and overall moisture management capability, characterizing the moisture transport ability of fabric, are the core parameters for evaluating the moisture absorbing and quick-drying properties of fabric substrate. As presented in Fig. 4a and Table S1, the double-side jacquard fabric exhibits excellent unidirectional moisture index (317.5%) slightly higher than that of cotton knitted fabrics (300.1%) and much better than that of woven and non-woven fabrics (168.8%, 140%, and − 30.2%, respectively). Meanwhile, the double-side jacquard fabric substrate demonstrates a dazzling liquid water dynamic transfer index (0.79), far superior to other four substrates (0.52, 0.35, 0.21, and 0.001, respectively). The double-side jacquard fabric exhibits such excellent moisture absorbing and quick-drying properties, which is attributed to its unique single-gradient infiltration structure. Specifically, the double jacquard fabric is composed of a surface hydrophilic layer (polybutylene terephthalate polyester, PBT) and a inner hydrophobic layer (polyethylene/polypropylene, PE/PP). Through loop knitting technology, the double-side jacquard fabric with a unilateral mesh structure has been fabricated. (The details of fabrication technology are shown in Methods and Fig. S16.) As a proof-of-concept, sweaty skin covered with the double-side jacquard fabric gets dry rapidly within minutes, demonstrating the outstanding moisture-wicking and quick-drying properties of double-side jacquard fabric (Figs. 4b and S17). The results prove that the double-side jacquard fabric is the best candidate substrate material for fabricating the meta-fabric.

In order to intuitively observe the directional moisture transport on the surface of double-side jacquard fabric, a moisture transferring test of fluorescent droplet (150 µL) on both sides of the double-side jacquard fabric under UVC environment was designed. When the droplet is dropped on the hydrophilic side (PBT), it rapidly penetrates the fabric and spreads to form a large wetted area quickly (≈ 0.5 s), as shown in Fig. 4c, d and Movies S1, S2. Conversely, when the droplet dripped on the hydrophobic side (PE/PP), it displays a slow penetration process and forms a wetting area on the hydrophilic side (Fig. 4e, f and Movies S3, S4). Benefiting the single-gradient infiltration structural of the double-side jacquard fabric, the meta-fabric exhibits a unique differential capillary effect. Specifically, the moisture absorbed by the hydrophilic layer of the double-side jacquard fabric can be captured by the ionic hydrogel under the synergistic effect of supramolecular interaction and osmotic pressure difference. Most importantly, this structure permits the moisture evaporated by the ionic hydrogel cannot diffuse into the external environment through the hydrophobic layer. Therefore, the meta-fabric can ensure a dry and comfortable skin environment at all times. Meanwhile, the double-side jacquard fabric substrate can keep the moisture inside the ionic hydrogel, thus prolonging the working time of the meta-fabric (> 7 days, 25 °C, Fig. 4g).

In order to clarify the mechanism of the differential capillary effect, a simplified model is used to describe the moisture transfer process in the meta-fabric (Fig. 4h). When moisture vapor generated by the human skin comes the interfacial hydrophobic layer, the moisture are transferred upward along the micro-channels inside PP/PE under the Laplace force [59, 60].

1 $$ P = \frac{4\gamma \cos \theta }{D} $$

where γ is the liquid–gas interfacial tension, *θ* is the contact angle on the fiber surface, and *D* is the pore size between fibers. Once the droplet contacts the meta-fabric between the hydrophobic layer and the hydrophilic layer, the sweat droplet is subjected to two Laplace pressure *P*_1_ and *P*_2_ in the same direction, with the resultant sucking force (Δ*P*) in the up direction.

2 $$ \Delta P = \frac{{4\gamma \cos \theta_{2} }}{{D_{2} }} - \frac{{4\gamma \cos \theta_{1} }}{{D_{1} }} = P_{2} + \left| {P_{1} } \right| $$

Therefore, once sweat comes into contact with the hydrophobic layer of the meta-fabric, the Laplace pressure generated continuously push the droplets toward the hydrophilic layer. Namely, this differential capillary effect with gravity-defying liquid transportation is the essential mechanism for maintaining the dry wearing interface. In addition, the PAAM-LiCl ionic hydrogel is rich in hydrophilic groups such as amide (–CONH_2_) and carboxyl groups (–COOH), which can produce supermolecular crosslinking interaction with the moisture captured by the hydrophilic layer. This interaction can effectively induce moisture diffusion from the hydrophilic layer to the ionic hydrogel. Meanwhile, the high concentration of salt solution in the ionic hydrogel generates a concentration pressure difference with the hydrophilic layer, further promoting the trend of moisture diffusion toward the ionic hydrogel. Therefore, the ionic hydrogel can continuously replenish moisture from the external environment during the wearing process to maintain the stability of its electrical properties. Interestingly, the moisture evaporated from the ionic hydrogel can be captured by the hydrophilic layer and diffuse downward. When the moisture reaches the meta-fabric between the hydrophilic and hydrophobic layers, it is repelled by an upward Laplace force, preventing it from further diffusing into the hydrophobic layer (Eq. 2). Therefore, a certain level of moisture retention has been dynamic achieved, enabling the meta-fabric to maintain stable performance for a long time. In addition, our fabrication strategy can realize the function of “hitting three birds with one stone.” Besides maintaining the dryness wearing experience and long working time, our meta-fabric also provides a cool-touch sensation because of the PE fibrous yarns in the hydrophobic layer (Fig. 4i). With high crystallization and orientation, PE fibers allow the external heat to dissipate quickly along the fiber axis, which can be proved by the instantaneous heat flow (Q-Max). As a key factor to evaluate the cooling sensing property of fabrics, Q-Max = *λ* × *T*, where *λ* is thermal conductivity and *T* is test temperature. One can see that the corresponding Q-Max data of our double-side knitted fabric can reach 5.3 W cm^−2^, nearly 35-folds than standard coolness value of clothing (0.15 W cm^−2^), indicating the merits of the structure of the meta-fabric (Fig. 4j).

### Grip Strength Visualization Rehabilitation Training Gloves

**Fig. 5:**
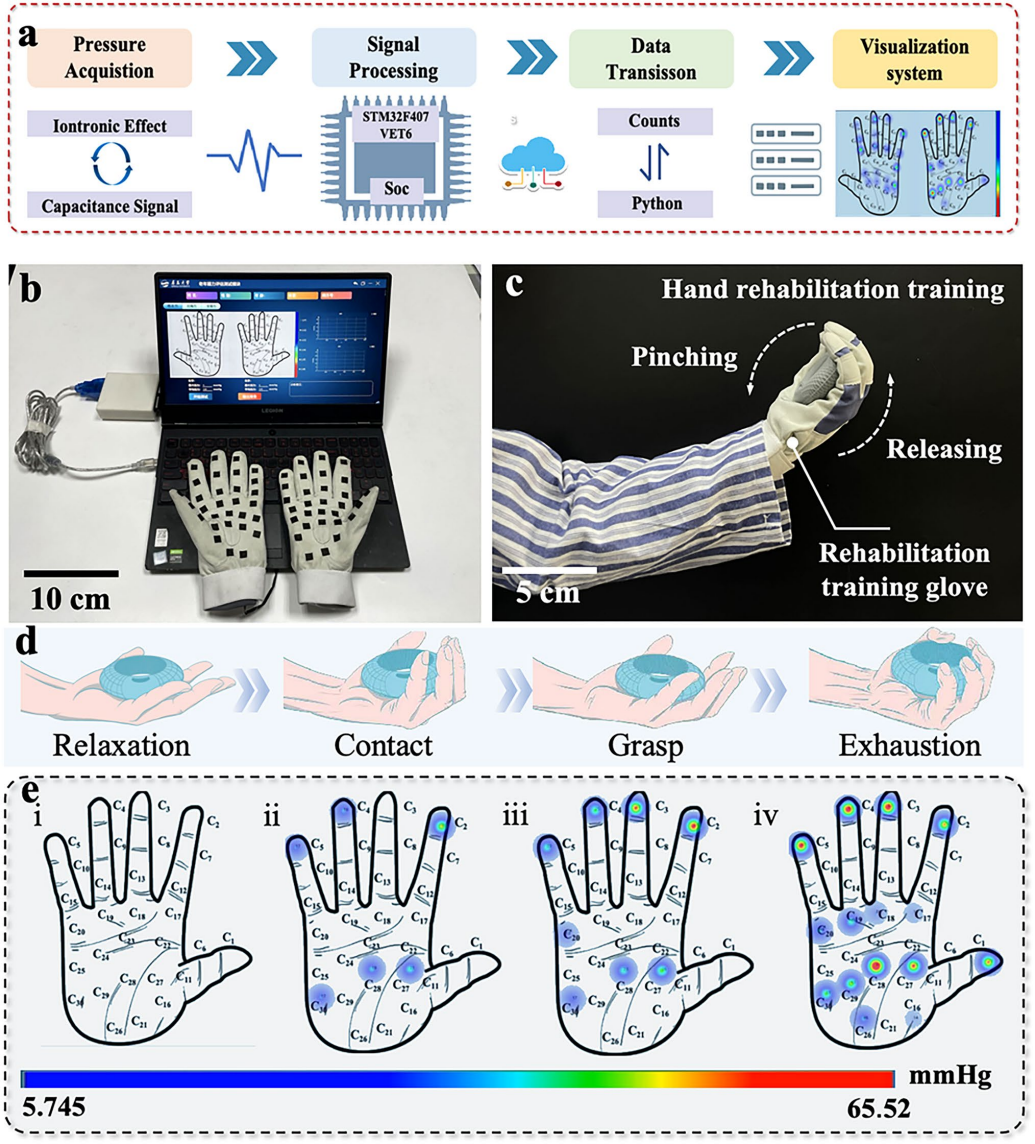
Construct and display of the grip strength visualization rehabilitation training system. **a** Components of the grip strength visualization rehabilitation training system: a pair of grip strength training glove, data analysis module and terminal display module.** b** Image of the grip strength visualization rehabilitation training system. **c** Image of the grip strength test (left hand). **d, e** Schematic of the four characteristic stages during grip strength training and corresponding visualization grip heat-map

Patients with spasticity often suffer from continuous muscle tension, which prevents the muscles from contracting and relaxing normally. Grip strength training can help patients gradually prompt muscle relaxation, so that their restore normal muscle function. Therefore, a smart ward based on grip strength visualization rehabilitation training system was proposed to verify the potential of our device in the field of rehabilitation. The system contains a pair of grip strength training gloves (30 sensing units per glove), a data acquisition board module, data analysis modules and terminal display modules (Figs. 5a, b and S18, S19). To demonstrate the advancement of our system more intuitively, the volunteer wearing training gloves conduct continuous grip strength training tests (Fig. 5c). During this process, four characteristic phases of grip strength training (relaxation, contact, grasp and exhaustion stage, respectively) were used to demonstrate the visualization function (Fig. 5d). In the relaxation stage, the heat-map of the hand does not show any pressure fluctuations (Fig. 5e(i)). Once the hand starts to flex, the squeezing effect generated by the palm joint and the grip ball stimulates the tactile sensorimotor interface at the corresponding position to generate electrical signals. Therefore, the heat-map of palm pressure begins to show a pressure distribution (contact stage, Fig. 5e(ii)). Notably, the color of the pressure heat-map is mainly cyan at this time (max pressure < 8.52 mmHg) due to the amplitude of hand muscle contraction being relatively small. As the degree of palm flexion increases, the squeezing effect between the joint and the grip ball becomes intense, leading to an increase in the capacitance excited by the tactile sensing interface. Therefore, the heat-map of pressure shows obvious red areas (> 61.87 mmHg), which proves that the muscle contraction of the joints at this position has intensified (grasp stage, Fig. 5e(iii)). Finally, when the tester reaches the exhaustion stage, the red area of the hand heat-map peaks. This result indicates that the hand multi-muscle group contraction reaches the extreme and produces the maximum grip strength (Fig. 5e(iv) and Movie S5). Through the above process, patients can visually understand the dilation and contraction ability of their hand muscles and conduct targeted training for certain muscles, improving the efficiency of rehabilitation training and accelerating the recovery process.

### Motor Balance Visualization Rehabilitation Training Carpet

**Fig. 6:**
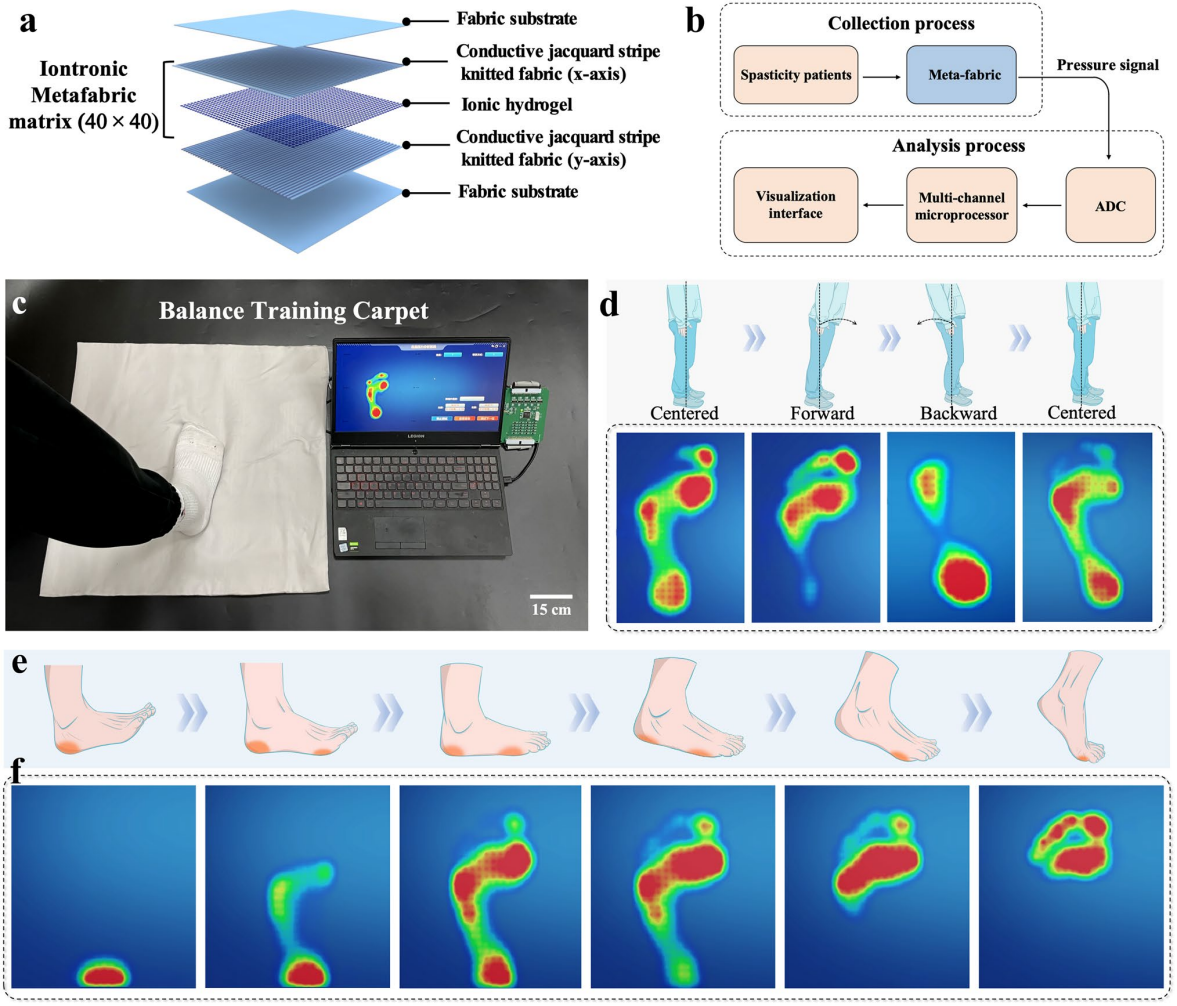
Construct and display of the motor balance visualization rehabilitation training system. **a** Structural schematic diagram of the motor balance carpet, including 40 × 40 iontronic meta-fabric matrix. **b** Block diagram of the meta-fabric visualization motor balance training.** c** Image of the motor balance visualization rehabilitation training carpet. **d** Pressure distribution heat-map during standing in three situations: forward tilt, centered, and backward tilt. **e**, **f** Pressure distribution heat-map of a complete gait cycle from foot-following to forefoot-strike-off

In addition to muscle tension, involuntary muscle contractions can cause balance disorders, increasing the risk of secondary injuries to patients. It is delighted to see that the patients can learn how to adjust their posture through foot balance training, thus improving the movement stability. In this case, a balance training carpet (500 × 500 mm^2^) has been assembled with a 40 × 40 tactile sensing array based on the Cortex-M4 core processor to achieve high-resolution visual rehabilitation training for motor balance (Figs. 6a and S20, the fabricating details seen in Methods). In order to minimize the number and complexity of electrode array wiring, a multiplexing wiring method was adopted to construct the circuit system (Fig. 6b, c). According to the physical structures of the foot, three main areas of the foot (forefoot, midfoot, and heel) have been tested and analyzed. Due to the high tactile sensing resolution of the interfaces, the pressure distribution in these three areas can be captured along with the change of posture. As shown in Fig. 6d and Movie S6, when a volunteer stands in a centered position, the biomechanical features lead to less weight-bearing in the midfoot area, while the forefoot and heel are the main weight-bearing areas. Particularly, the forefoot bears the greatest load. Once the volunteer leans forward slightly, the overall pressure on the forefoot increases due to the forward shift of the body’s center of gravity. Conversely, with a slight tilt of the body, the plantar pressure is mainly concentrated on the heel. These trends can be well captured by the corresponding plantar pressure heat-map of the balance training carpet. Namely, the carpet can accurately identify differences in human posture, as well as timely detect abnormal foot pressure in patients. In addition, a complete gait cycle test has also been designed based on the carpet, and the plantar pressure changes throughout the gait behavior of the volunteer can be reflected by the corresponding visual pressure heat-map (Fig. 6e, f and Movie S7). This system is conductive to real-time monitoring and analyzing the motor stability of patients, enabling doctors to timely understand the patients accurately physical condition and improve the efficiency of rehabilitation treatment.

## Conclusions

In summary, spasticity visualization rehabilitation training systems based on skin-friendly large matrix iontronic meta-fabric and well-trained data analysis modules have been proposed. The meta-fabric exhibits remarkable tactile sensitivity and high-sensing resolution (50 Pa or 0.058%) over a broad pressure range of up to 270 kPa. Particularly, it reveals a “hitting three birds with one stone” property (dryness wearing experience, long working time and cooling sensing, respectively). These merits are mainly attributed to the differential capillary effect through precise designing the fabric substrate with a hydrophilic–hydrophobic double-layer structure. To demonstrate the application potential of our meta-fabric in the medical field, the visualization grip strength training gloves and motor balance training carpet have been fabricated. Thanks to the advantages of the meta-fabric, these devices can display real time the exact pressure distribution resulting from human motion during the process of precise rehabilitation training. We believe that our devices can provide a more intelligent, personalized and comfortable healthcare experience for patients suffering from spasticity, and it is expected to be a good candidate for the next-generation assistive technique for closed-loop spasticity rehabilitation training.

## Supplementary Information

Below is the link to the electronic supplementary material.

Supplementary file1 (DOCX 1947 KB)

Supplementary file2 (MP4 6126 KB)

Supplementary file3 (MP4 3537 KB)

Supplementary file4 (MP4 4061 KB)

Supplementary file5 (MP4 5324 KB)

Supplementary file6 (MP4 12323 KB)

Supplementary file7 (MP4 10674 KB)

Supplementary file8 (MP4 6812 KB)

## Data Availability

All code supporting this study’s findings is available from the corresponding authors upon request.
