# Supplementary material for: Skin-Friendly Large Matrix Iontronic Sensing Meta-Fabric for Spasticity Visualization and Rehabilitation Training via Piezo-Ionic Dynamics
Source: Nanomicro Lett. 2024 Dec 19;17:90. doi: 10.1007/s40820-024-01566-3 (PMC11655817; doi:10.1007/s40820-024-01566-3)
Supplement: Supplementary file 1 — Supplementary file1 (DOCX 1947 KB) [file 40820_2024_1566_MOESM1_ESM.docx]

Supporting Information for

**Skin-Friendly Large Matrix Iontronic Sensing Meta-Fabric for Spasticity Visualization and Rehabilitation Training via Piezo-Ionic Dynamics**

Ruidong Xu^1,#^, Tong Xu^1,#^, Minghua She^1^, Xinran Ji^2^, Ganghua Li^1^, Shijin Zhang^1^, Xinwei Zhang^1^, Hong Liu^1,^*, Bin Sun^3,^*, Guozhen Shen^4,^*, Mingwei Tian^1,^*

^1^Research Center for Intelligent and Wearable Technology, College of Textiles and Clothing, State Key Laboratory of Bio-Fibers and Eco-Textiles, Collaborative Innovation Center for Eco-textiles of Shandong Province and the Ministry of Education, Intelligent Wearable Engineering Research Center of Qingdao, Qingdao University, Qingdao 266071, P. R. China

^2^Academy of Arts & Design of Qingdao University, Qingdao 266071, P. R. China

^3^College of Electronics and Information, Qingdao University, Qingdao 266071, P. R. China

^4^School of Integrated Circuits and Electronics, Beijing Institute of Technology, Beijing 100081, P. R. China

^#^Ruidong Xu and Tong Xu contributed equally to this work.

*Corresponding authors. E-mail: [lh1221@qdu.edu.cn](mailto:lh1221@qdu.edu.cn) (Hong Liu); [qdusun@qdu.edu.cn](mailto:qdusun@qdu.edu.cn) (Bin Sun); [gzshen@bit.edu.cn](mailto:gzshen@bit.edu.cn) (Guozhen Shen); [mwtian@qdu.edu.cn](mailto:mwtian@qdu.edu.cn) (Mingwei Tian)

**Supplementary Figures and Tables**


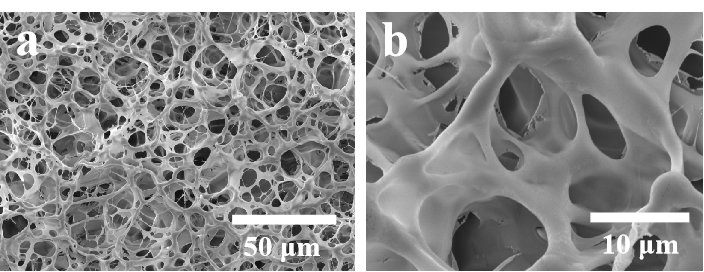


**Fig. S1** Micro-structure of the dome-shaped ionic hydrogel


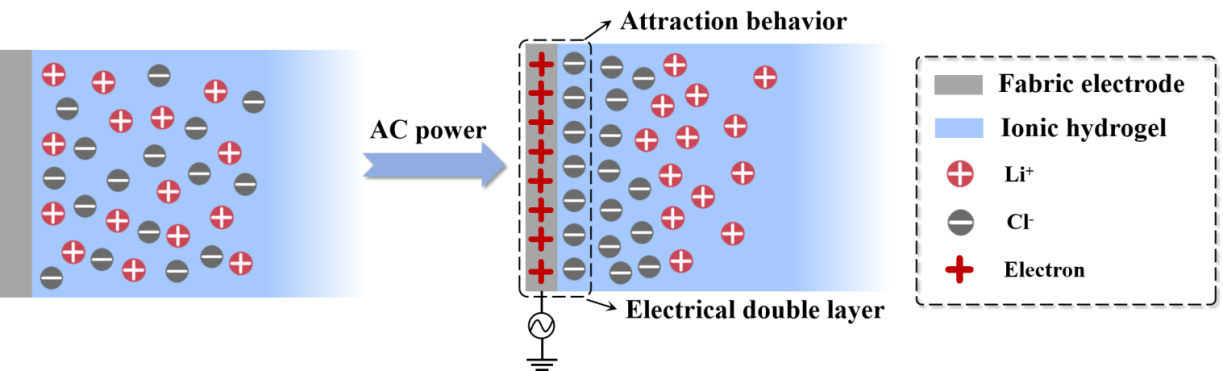


**Fig. S2** The formation mechanism of the electric double layer (EDL)

According to our previous work, we have calculated the capacitance of the electric double layer (C_EDL_) as follow:

$\frac{1}{C_{EDL}}=\frac{1}{C_{C}}+\frac{1}{C_{D}}$ (S1)

where *C_C_* is the capacitance value of the compact layer, *C_D_* is the capacitance value of the diffusion layer. When high frequency AC power is applied to the electrodes, the capacitance value of the diffusion layer (*C_D_*) is much larger than the capacitance value of the compact layer (*C_C_*). Therefore, the equation 1 can be expressed as:

$C_{EDL}\approx C_{C}$ (S2)

Due to the strong force between the electrode and the ion, the compact layer can be equivalent to a parallel plate capacitor. Therefore, the capacitance value of the compact layer (*C_C0_*) per unit area can be expressed:

$C_{C0}=\frac{\varepsilon_{\text{r}}\varepsilon_{0}}{d}A_{0}$ (S3)

Where *ε_r_* and *ε_0_* are dielectric constant and vacuum dielectric constant, respectively. *d* is the distance of the compact layer. *A*_0_ is the contact area between the ionic hydrogel and electrode. Therefore, the capacitance of double electric layer (*C_EDL0_*) per unit area can be expressed as follow:

$C_{EDL0}\approx\frac{\varepsilon_{r}\varepsilon_{0}}{d}A_{0}\approx$constant (S4)


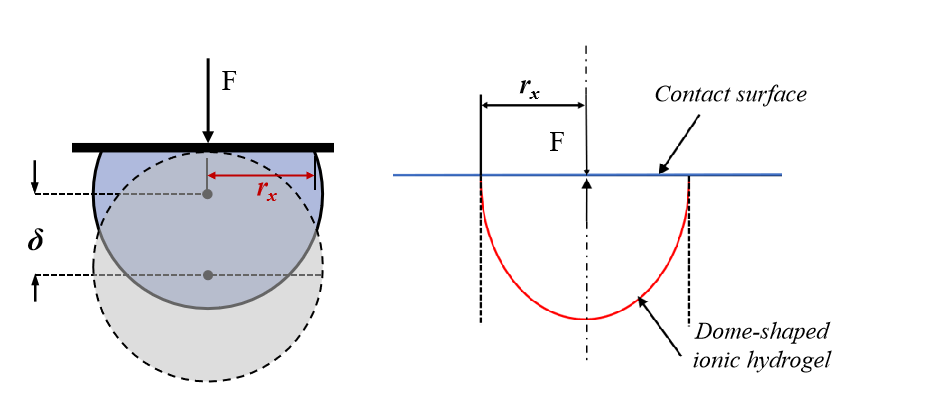


**Fig. S3** The dome-shaped ionic hydrogel area variation under external load pressure

According to Hertz contact theory when the external pressure is applied to the knitted fabric electrode, the deformation of single ionic hydrogel can be expressed as

$r_{x}=\left(\frac{3FR\left(1-\nu^{2}\right)}{4E}\right)^{\frac{1}{3}}$ (S5)

Where *R* is the diameter of the dome-shaped ionic hydrogel, *v* and *E* is the Poisson’s ration and elastic modulus of the dome-shaped ionic hydrogel, respectively. *F* is the external pressure loading. Meanwhile, the total normal deflection of the dome-shaped ionic hydrogel can be expressed as

$\delta=\left(\frac{9\left[F\left(1-\nu^{2}\right)\right]^{2}}{16\left(RE\right)^{2}}\right)^{\frac{1}{3}}$ (S6)

Therefore, the contact area of the single dome-shaped ionic hydrogel can be expressed as

$S_{X}=\pi\left(\frac{3FR\left(1-\nu^{2}\right)}{4E}\right)^{\frac{2}{3}}$ (S7)

Further, the overall contact area of the dome-shaped active layer of our programmable tactile sensoriomotor interface can be expressed as

$S_{X}=N\pi\left(\frac{3FR\left(1-\nu^{2}\right)}{4E}\right)^{\frac{2}{3}}$ (S8)

Where *N* is the number of the dome-shaped ionic hydrogel. Therefore, under external pressure stimuli, the capacitance of our interface can be expressed as

$C_{EDL1}=N\pi\frac{\varepsilon_{r}\varepsilon_{0}}{d}\left(\frac{3FR\left(1-\nu^{2}\right)}{4E}\right)^{\frac{2}{3}}$ (S9)


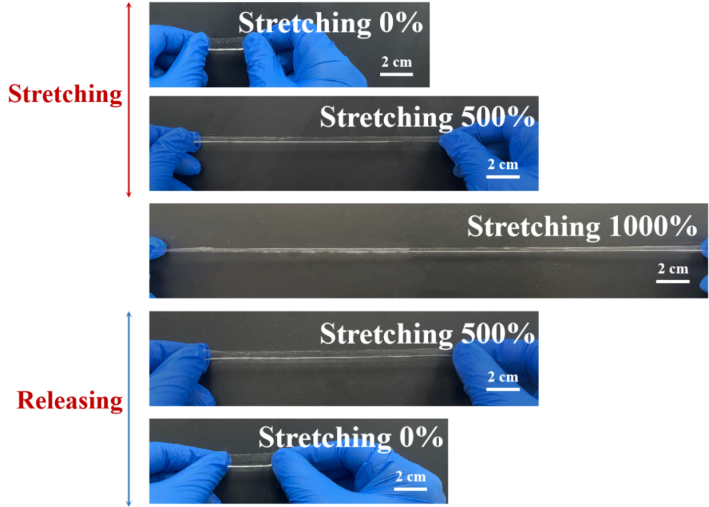


**Fig. S4** The excellent mechanical property of the PAAM-LiCl ionic hydrogel

The components of the tactile sensorimotor interface (the weft-knitted double-sided jacquard fabric and ionic hydrogel) are all soft matter. Notably, PAAM-LiCl hydrogel can endure 1000% stretching and has negligible hysteresis. This excellent mechanical performance stems from its unique 3D porous structure and the large amount of water between the polymer chains [S1-S7].


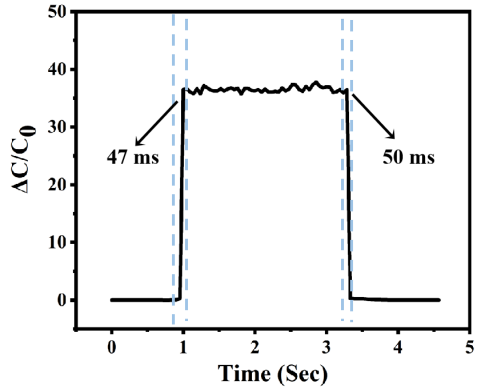


**Fig. S5** The response and release time of the tactile sensorimotor interface


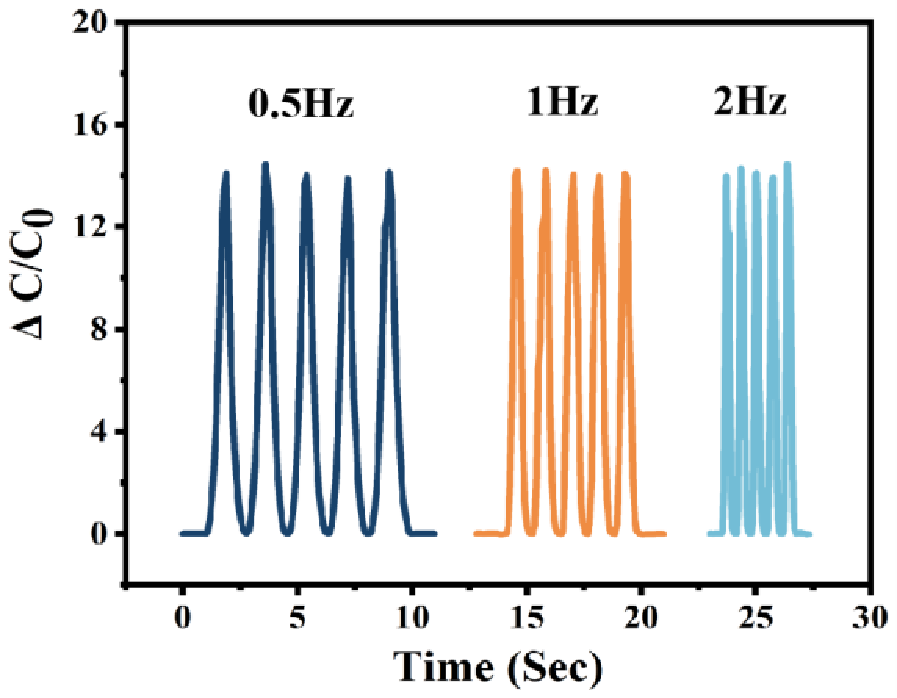


**Fig. S6** Tactile sensing stability test of our interface at different compression frequencies: 0.5, 1 and 2 Hz


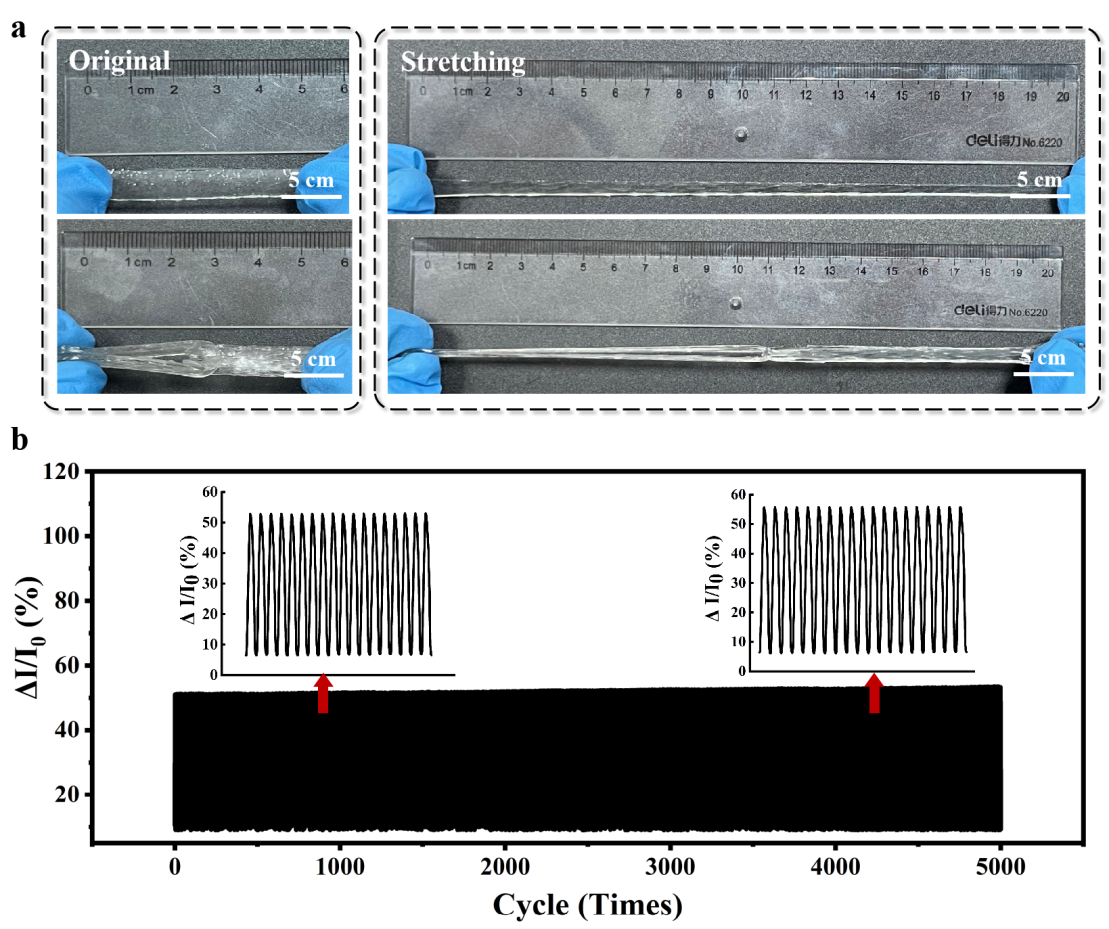


**Fig. S7** Characteristic of the ionic hydrogel. (**a**) Brilliant intrinsic flexibility of ionic hydrogel. (**b**) Stable electrical property of ionic hydrogel


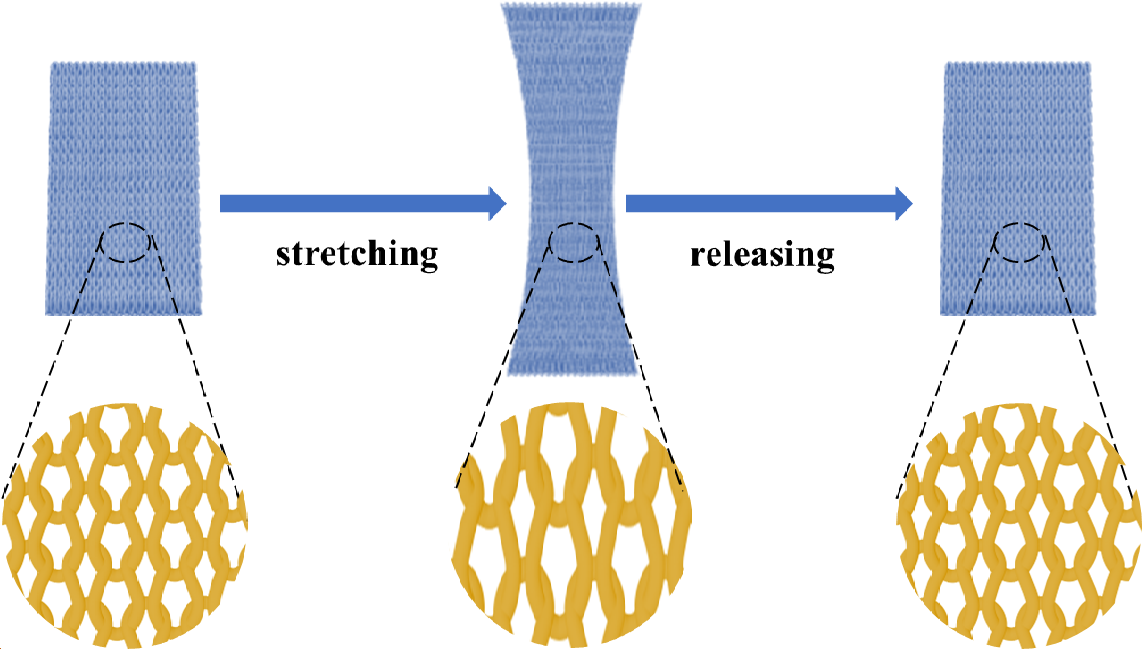


**Fig. S8** Resilience property of knitted fabric: unique loop structure


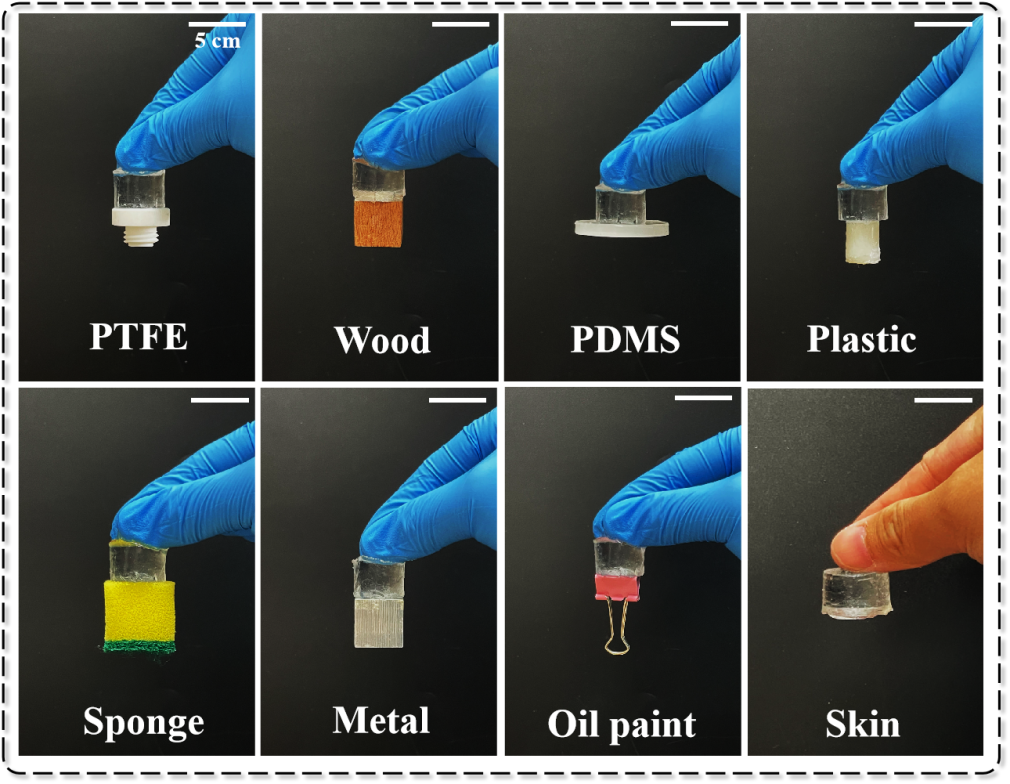


**Fig. S9** Illustration of adhesion property of the ionic hydrogel. Photographs of ionic hydrogel adhered on various polar substrates (PTFE, wood, PDMS, plastic, sponge, metal, oil paint and skin)


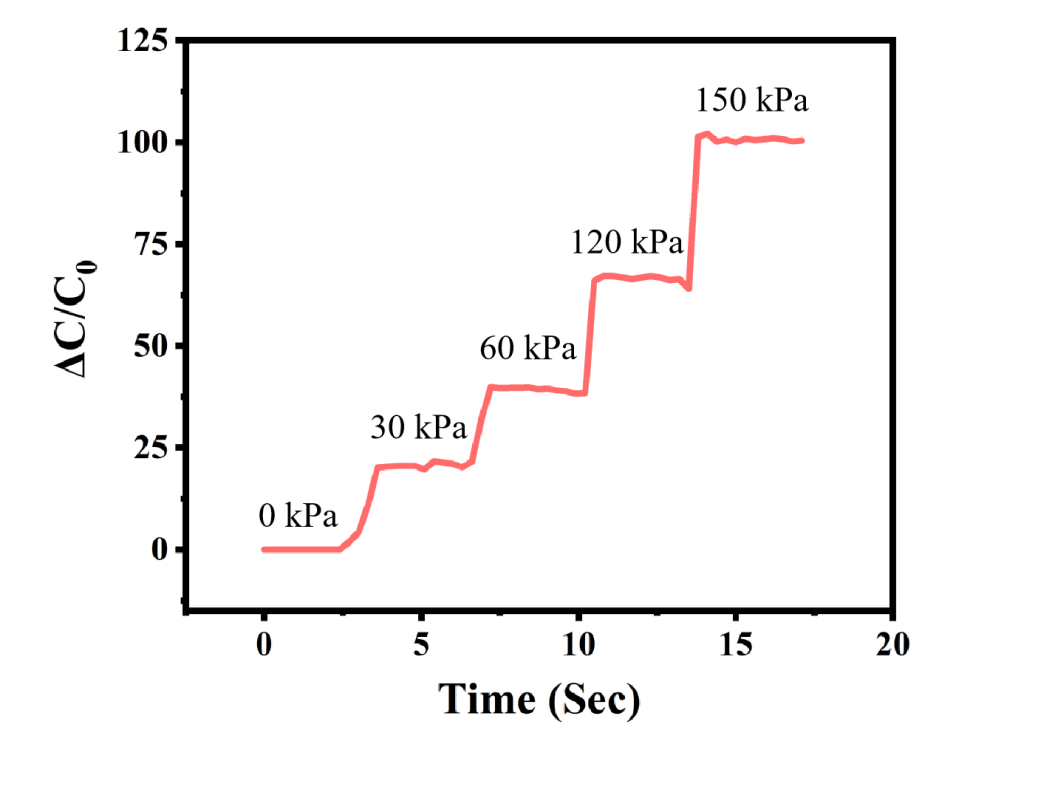


**Fig. S10** Excellent tactile sensing property of our interface under gradient pressure loading (0, 30, 60, 120, 150 kPa)


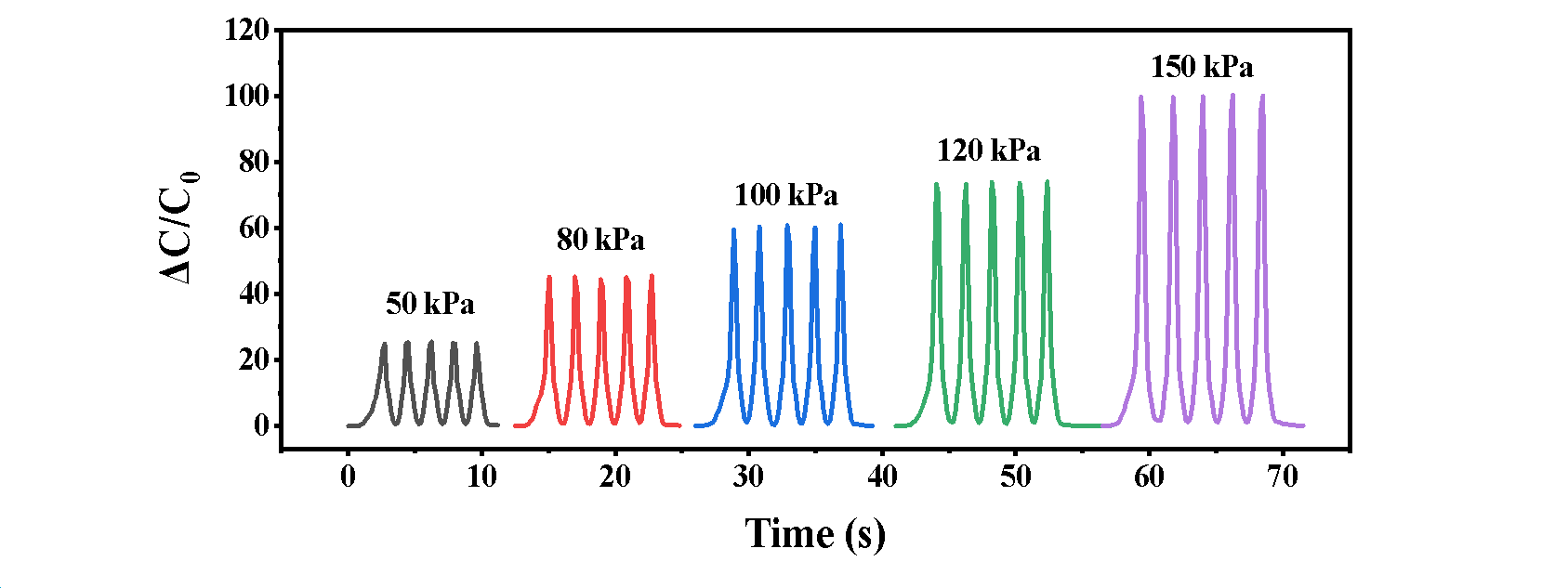


**Fig. S11** Stability property of our interface under different pressure loading: 50, 80, 100, 120, 150 kPa


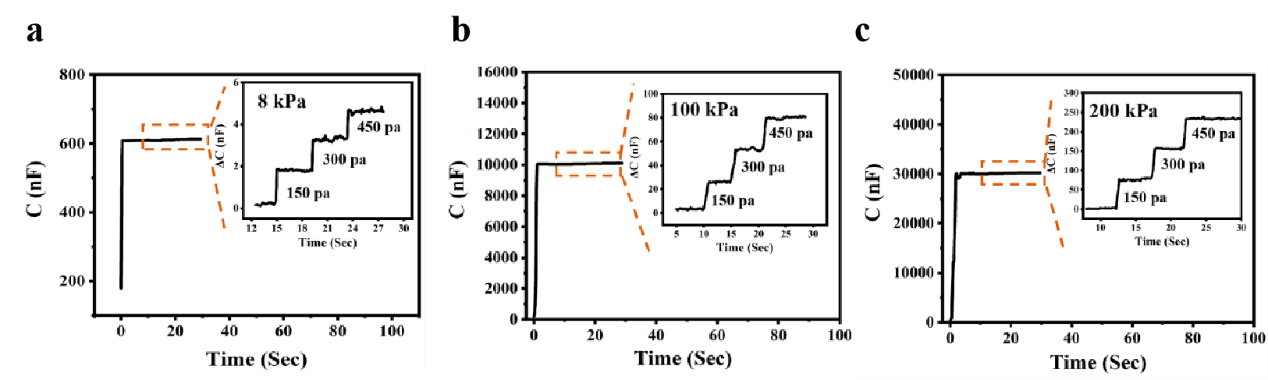


**Fig. S12** The high pressure resolution of the programmable tactile sensorimotor interface under different reference pressures of P=8, 100 and 200 kPa


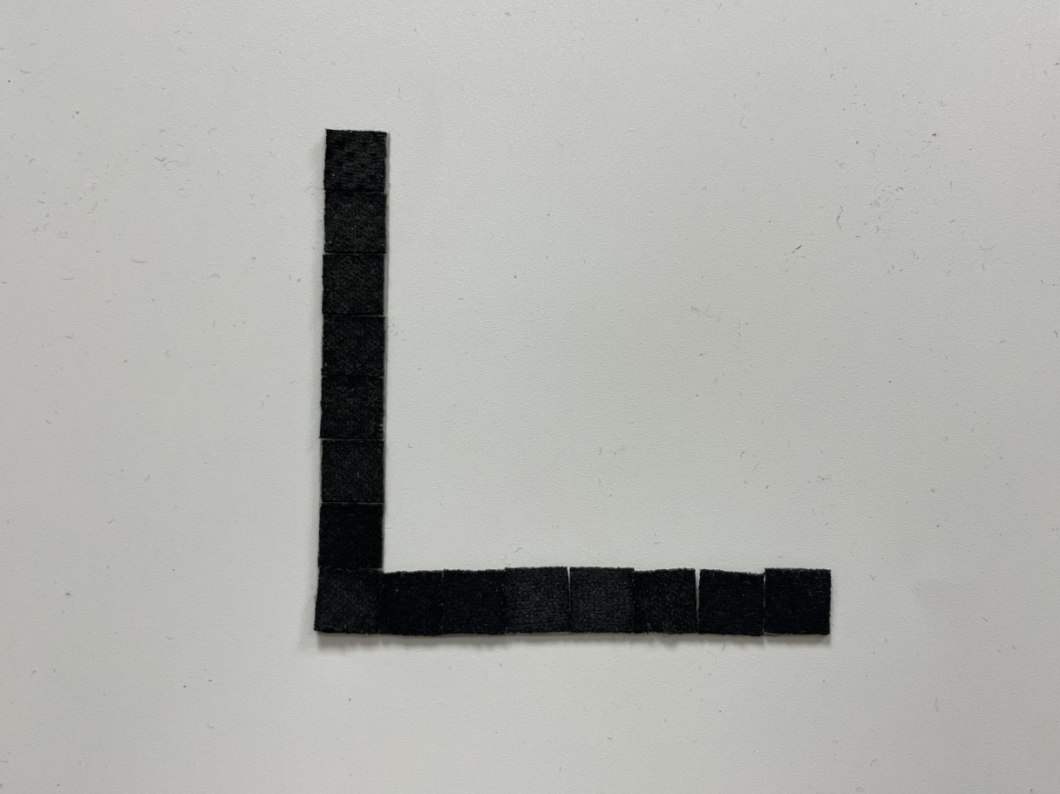


**Fig. S13** Image of L-shaped control positioning tactile interface


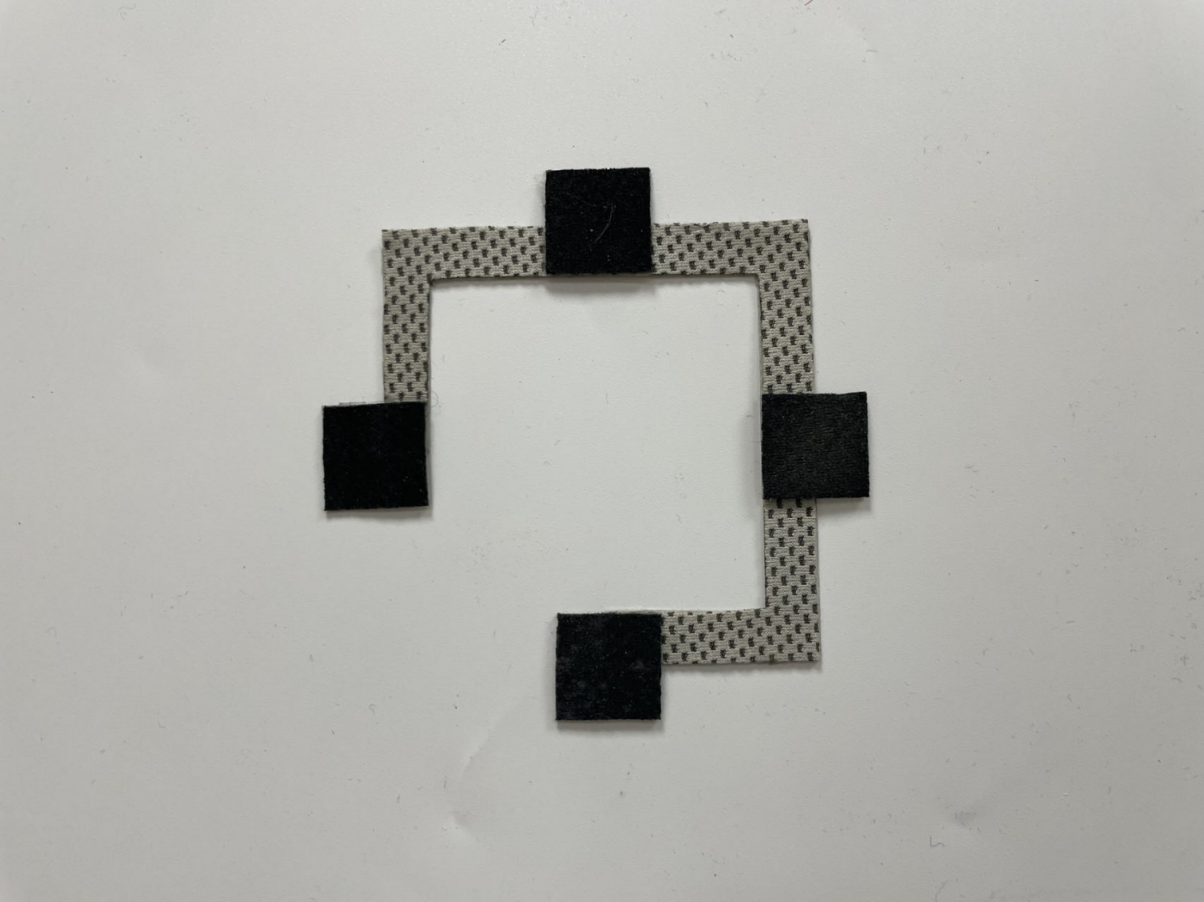


**Fig. S14** Image of direction control rotation


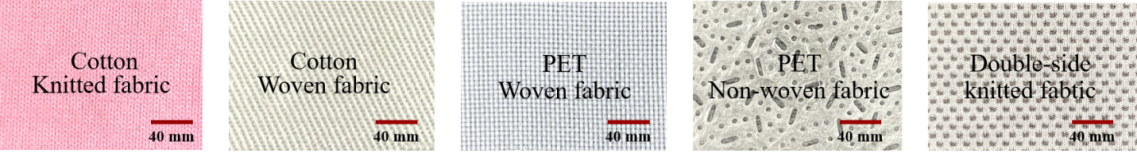


**Fig. S15** Images of five type fabric substrate materials: cotton knitted fabric, cotton woven fabric, PET woven fabric, PET non-woven fabric and double-side fabric, respectively


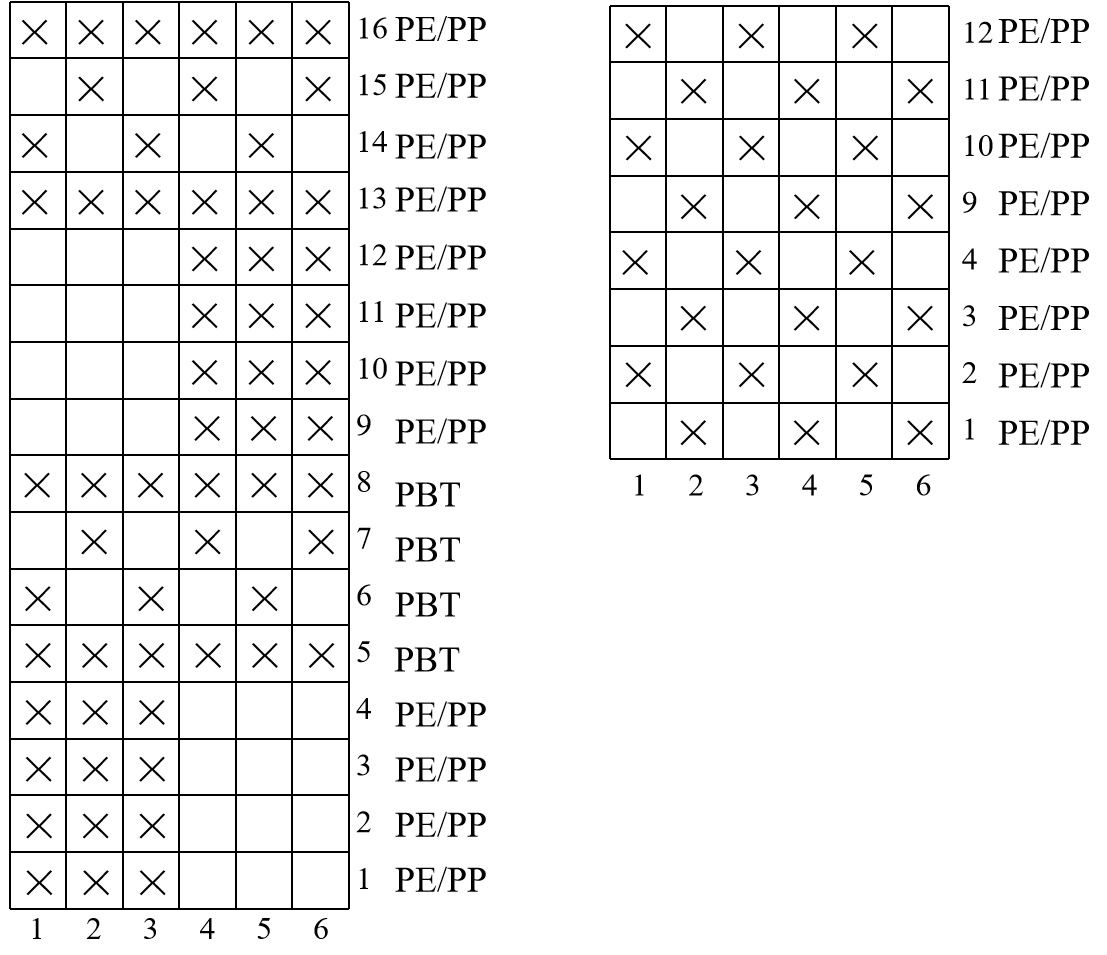


**Fig. S16** The pattern grid of the weft-knitted double-sided jacquard fabric


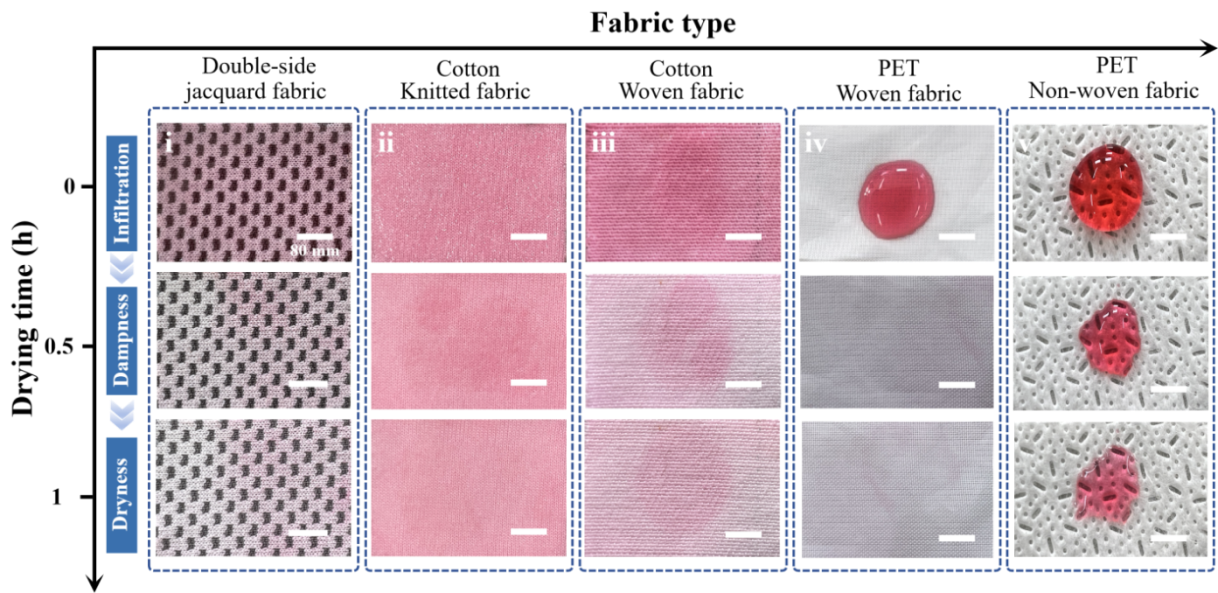


**Fig. S17** Drying process of different fabric substrate: double-side jacquard fabric (our work), cotton knitted fabric, cotton woven fabric, PET woven fabric and PET non-woven, respectively

The double-side jacquard fabric can achieve dryness in 1 hour (**Fig. S17, i**). Cotton knitted and woven fabric shows a similar hygroscopicity like our work. However, the drying process of cotton fabrics (knitted and woven) is slow, which is due to the fact that its single-side structure does not possess unidirectional moisture absorption property (**Fig. S17, ii**). Besides, the drying speed of cotton knitted fabric is better than cotton woven fabric, which is due to the loose knitted structure conductive to moisture diffusion (**Fig. S17 and iii)**. The PET woven fabric exhibits poor hygroscopicity compared to cotton fabrics. However, the shaped structure of the PET fibers (cross shape) can increase moisture evaporation area, which is attribute to moisture conduct and diffuse quickly. Therefore, the PET woven fabric exhibits quick-drying characteristics (**Fig. S17, iv**). In addition, the PET non-woven fabric shows hydrophobic property, which is due to it dense fiber network structure (**Fig. S17, v**).


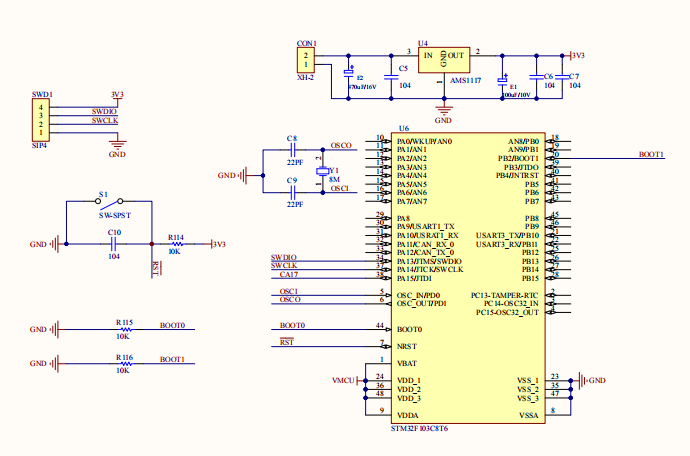


**Fig. S18** Schematic Diagram of the microcontroller circuit (MCU)


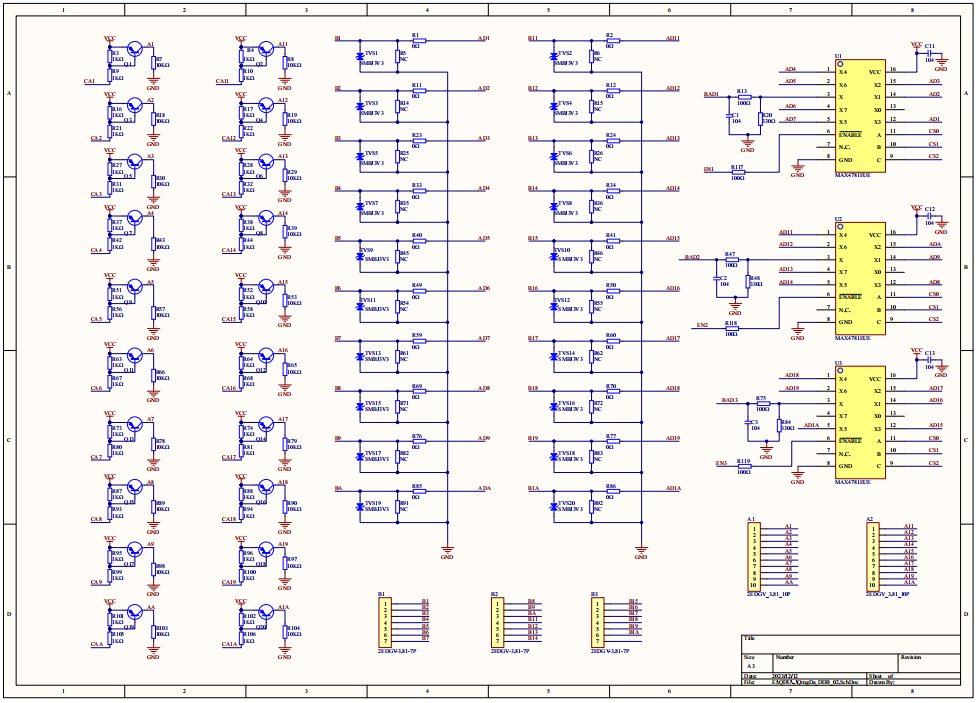


**Fig. S19** Schematic of multi-channel data acquisition board circuit


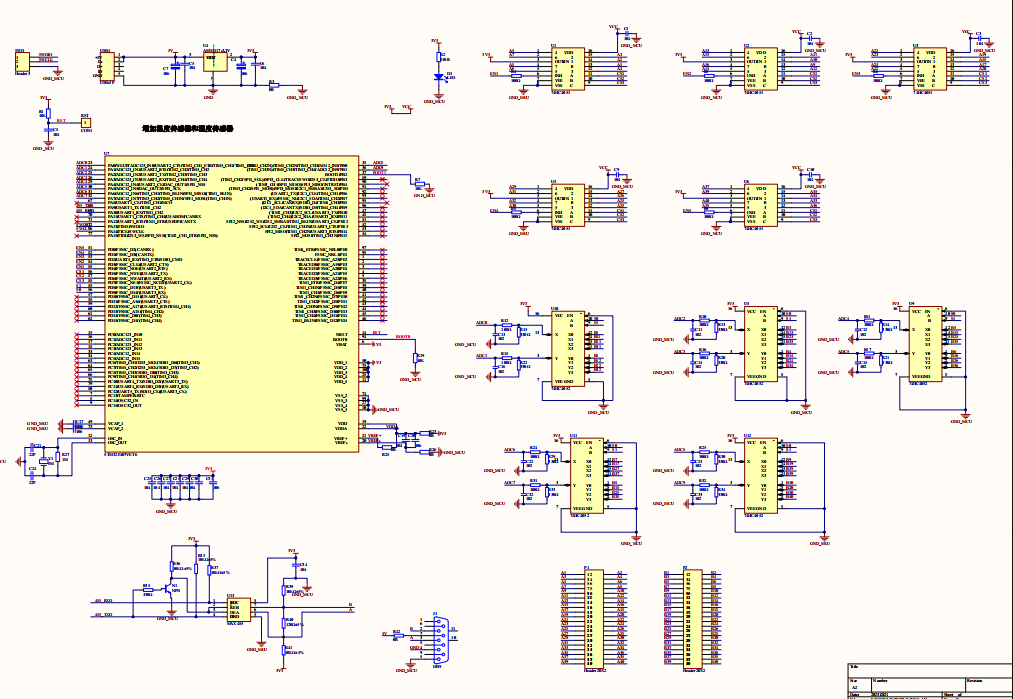


**Fig. S20** Schematic of multi-channel data acquisition board circuit

**Table S1** Sensitivity and detection range comparison of previously reported tactile sensors

| Structure | Maximum detection range (kPa) | Sensitivity (kPa^-1^) | References |
| --- | --- | --- | --- |
| **Bioinspired dome-shaped** | **270** | **6.47** | **This work** |
| Flat cylinder | 1 | 0.17 | [S8] |
| Spheres | 1.4 | 0.176 | [S9] |
| Self-patterned micro-architecture | 7 | 0.05 | [S10] |
| Plane | 15 | 0.6 | [S11] |
| Plane | 88 | 0.91 | [S12] |
| Plane | 4.5 | 0.877 | [S13] |
| Micro-lattices | 1.2 | 7.1 | [S14] |
| Spheres | 9 | 7.7 | [S15] |
| Capsule | 10 | 0.45 | [S16] |
| Plane | 50 | 0.127 | [S17] |
| Biomimetic skin-like | 150 | 0.131 | [S18] |
| Helix | 180 | 4.4 | [S19] |
| Porousness | 230 | 0.005 | [S20] |
| Plane | 240 | 0.192 | [S21] |
| Porousness | 250 | 0.136 | [S22] |
| Micro-spheres | 270 | 2.49 | [S23] |

**Table S2** Characterization indexes quickly-drying of five kinds of fabric

| Parameter  Sample | Surface wetting time (Sec) | Substrate wetting time (Sec) | Surface water absorption rate (%/s) | substrate water absorption rate (%/s) | Surface  water diffusion rate (mm/s) | Substrate water diffusion rate (mm/s) | Unidirectional moisture conductivity（%） | OMMC |
| --- | --- | --- | --- | --- | --- | --- | --- | --- |
| **Our work** | **2.808** | **2.56** | **39.3** | **57.9** | **5.15** | **5.41** | **317.5** | **0.79** |
| Cotton knitted fabric | 8.99 | 13.10 | 25.82 | 99.22 | 1.32 | 1.27 | 137.71 | 0.48 |
| Cotton woven fabric | 15.73 | 8.52 | 37.18 | 36.64 | 1.37 | 1.47 | 166.83 | 0.35 |
| PET woven fabric | 8.76 | 16.58 | 100.81 | 67.10 | 0.56 | 0.40 | 306.21 | 0.52 |
| PET non-woven fabric | 9.15 | 103.53 | 138.70 | 2.37 | 0.54 | 0.03 | -125.05 | 0.001 |

**Table S3** Characterization indexes quickly-drying of five kinds of fabric

| Parameter  Sample | Surface wetting time (Sec) | Substrate wetting time (Sec) | Surface water absorption rate (%/s) | substrate water absorption rate (%/s) | Surface  water diffusion rate (mm/s) | Substrate water diffusion rate (mm/s) | Unidirectional moisture conductivity（%） | OMMC |
| --- | --- | --- | --- | --- | --- | --- | --- | --- |
| **Our work** | **2.808** | **2.56** | **39.3** | **57.9** | **5.15** | **5.41** | **317.5** | **0.79** |
| Cotton knitted fabric | 8.99 | 13.10 | 25.82 | 99.22 | 1.32 | 1.27 | 137.71 | 0.48 |
| Cotton woven fabric | 15.73 | 8.52 | 37.18 | 36.64 | 1.37 | 1.47 | 166.83 | 0.35 |
| PET woven fabric | 8.76 | 16.58 | 100.81 | 67.10 | 0.56 | 0.40 | 306.21 | 0.52 |
| PET non-woven fabric | 9.15 | 103.53 | 138.70 | 2.37 | 0.54 | 0.03 | -125.05 | 0.001 |

**Supplementary References**

1. Y. S. Zhang, A. Khademhosseini, Advances in engineering hydrogels. Science **356**, 6337 (2017). [http://doi.org/10.1126/science.aaf3627](http://doi/org/10.1126/science.aaf3627)
2. H. Ke, L.P. Yang, M. Xie, Z. Chen, H. Yao et al., Shear-induced assembly of a transient yet highly stretchable hydrogel based on pseudopolyrotaxanes. Nat. Chem. **11**, 470-477 (2019). <http://doi.org/10.1038/s41557-019-0235-8>
3. F. G. Downs, D. J. Lunn, M. J. Booth, J. B. Sauer, W. J. Ramsay et al., Multi-responsive hydrogel structures from patterned droplet networks. Nat. Chem.**12**, 363-371 (2020). <http://doi.org/10.1038/s41557-020-0444-1>
4. D. R. Griffin, M. M. Archang, C.H. Kuan, W. M. Weaver, J. S. Weinsteinety et al., Activating an adaptive immune response from a hydrogel scaffold imparts regenerative wound healing. Nat. Mater. **20**, 560-569 (2021). <http://doi.org/10.1038/s41563-020-00844-w>
5. A. V. Salvekar, W. M. Huang, R. Xiao, Y. S. Wong, S. S. Venkatraman et al., Water-responsive shape recovery induced buckling in biodegradable photo-cross-linked poly(ethylene glycol) (PEG) hydrogel. Acc. Chem. Res. **50**, 141-150 (2017). <http://doi.org/10.1021/acs.accounts.6b00539>
6. Y. Zhou, C. Wan, Y. Yang, H. Yang, S. Wang et al., Highly stretchable, elastic, and ionic conductive hydrogel for artificial soft electronics. Adv. Funct. Mater. **29**, 1806220 (2019). <http://doi.org/10.1002/adfm.201806220>
7. C.C. Kim, H.H. Lee, K. H. Oh, J.Y. Sun, Highly stretchable, transparent ionic touch panel. Science **353**, 682-687 (2016). <http://doi.org/10.1126/science.aaf8810>
8. Z. Lei, Q. Wang, S. Sun, W. Zhu, P. Wu, A bioinspired mineral hydrogel as a self-healable, mechanically adaptable ionic skin for highly sensitive pressure sensing. Adv. Mater. **29**, 1700321 (2017). <https://doi.org/10.1002/adma.201700321>
9. Y. Tai, M. Mulle, I. Aguilar Ventura, G. Lubineau, A highly sensitive, low-cost, wearable pressure sensor based on conductive hydrogel spheres. Nanoscale **7**, 14766-14773 (2015). <https://doi.org/10.1039/c5nr03155a>
10. G. Ge, Y. Zhang, J. Shao, W. Wang, W. Si et al., Stretchable, transparent, and self-patterned hydrogel-based pressure sensor for human motions detection. Adv. Funct. Mater. **28**, 1802576 (2018). <https://doi.org/10.1002/adfm.201802576>
11. Z. Huang, X. Feng, T. Zhang, Z. Liu, B. Zhu et al., Highly stretchable hydrogels for sensitive pressure sensor and programmable surface patterning by thermal bubble inkjet technology. J. Appl. Polym. Sci. **137**, 49146 (2020). <https://doi.org/10.1002/app.49146>
12. H. Ding, Z. Wu, H. Wang, Z. Zhou, Y. Wei et al., An ultrastretchable, high-performance, and crosstalk-free proximity and pressure bimodal sensor based on ionic hydrogel fibers for human-machine interfaces. Mater. Horiz. **9**, 1935-1946 (2022). <https://doi.org/10.1039/d2mh00281g>
13. S. Feng, Q. Li, S. Wang, B. Wang, Y. Hou et al., Tunable dual temperature-pressure sensing and parameter self-separating based on ionic hydrogel via multisynergistic network design. ACS Appl. Mater. Interfaces **11**, 21049-21057 (2019). <https://doi.org/10.1021/acsami.9b05214>
14. M. Yue, Y. Wang, H. Guo, C. Zhang, T. Liu, 3D reactive printing of polyaniline hybrid hydrogel microlattices with large stretchability and high fatigue resistance for wearable pressure sensors. Compos. Sci. Technol. **220**, 109263 (2022). <https://doi.org/10.1016/j.compscitech.2022.109263>
15. H. Zhou, M. Wang, X. Jin, H. Liu, J. Lai et al., Capacitive pressure sensors containing reliefs on solution-processable hydrogel electrodes. ACS Appl. Mater. Interfaces **13**, 1441-1451 (2021). <https://doi.org/10.1021/acsami.0c18355>
16. N. Kallingal, M. R. Maurya, M. S. Sajna, H. C. Yalcin, H. M. Ouakad et al., A highly sensitive wearable pressure sensor capsule based on PVA/Mxene composite gel. 3 Biotech. **12**, 171 (2022). <https://doi.org/10.1007/s13205-022-03221-3>
17. Z. Qin, X. Sun, Q. Yu, H. Zhang, X. Wu et al., Carbon nanotubes/hydrophobically associated hydrogels as ultrastretchable, highly sensitive, stable strain, and pressure sensors. ACS Appl. Mater. Interfaces **12**, 4944-4953 (2020). <https://doi.org/10.1021/acsami.9b21659>
18. S. Xia, Q. Zhang, S. Song, L. Duan, G. Gao, Bioinspired dynamic cross-linking hydrogel sensors with skin-like strain and pressure sensing behaviors. Chem. Mater. **31**, 9522-9531 (2019). <https://doi.org/10.1021/acs.chemmater.9b03919>
19. Y. Wu, Y. Liu, Y. Zhou, Q. Man, C. Hu et al., A skin-inspired tactile sensor for smart prosthetics. Sci. Robot. **3**, eaat0429 (2018). <https://doi.org/10.1126/scirobotics.aat0429>
20. E. S. Hosseini, M. Chakraborty, J. Roe, Y. Petillot, R. S. Dahiya, Porous elastomer based wide range flexible pressure sensor for autonomous underwater vehicles. IEEE Sens. J. **22**, 9914-9921 (2022). <https://doi.org/10.1109/jsen.2022.3165560>
21. H. Shi, I. González-Afanador, C. Holbrook, N. Sepúlveda, X. Tan, Soft pressure sensor for underwater sea lamprey detection. IEEE Sens. J. **22**, 9932-9944 (2022). <https://doi.org/10.1109/jsen.2022.3166693>
22. C.Y. Huang, G. Yang, P. Huang, J.M. Hu, Z.H. Tang et al., Flexible pressure sensor with an excellent linear response in a broad detection range for human motion monitoring. ACS Appl. Mater. Interfaces **15**, 3476-3485 (2023). <https://doi.org/10.1021/acsami.2c19465>
23. W. Chen, B. Wang, Q. Zhu, X. Yan, Flexible pressure sensors with a wide detection range based on self-assembled polystyrene microspheres. Sensors **19**, 5149 (2019). <https://doi.org/10.3390/s19235194>
